# Efficacy of Telemedical Interventional Management in Patients with Coronary Heart Disease Undergoing Percutaneous Coronary Intervention: Randomized Controlled Trial

**DOI:** 10.2196/63350

**Published:** 2025-10-20

**Authors:** Xiaofan Yu, Jiaoyu Cao, Jie Xu, Qizhi Xu, Hongwu Chen, Dongbiao Yu, Anping Ou, Yue Hu, Likun Ma

**Affiliations:** 1 Department of Cardiology, The First Affiliated Hospital of USTC, Division of Life Sciences and Medicine, University of Science and Technology of China, No. 17, Lujiang Road, Hefei, 230001, China, 86 13365741273, 86 055162284055; 2 Department of Cardiology, Centre for Leading Medicine and Advanced Technologies of IHM, The First Affiliated Hospital of USTC, Division of Life Sciences and Medicine, University of Science and Technology of China, Hefei, China

**Keywords:** telemedical interventional management, coronary heart disease, secondary prevention, percutaneous coronary intervention, efficacy, heart disease, patient management, remote, randomized controlled trial, China

## Abstract

**Background:**

Coronary heart disease (CHD) continues to be a leading cause of global morbidity and mortality, with patients undergoing percutaneous coronary intervention (PCI) facing a significant risk of recurrent cardiovascular events. While secondary prevention strategies, such as medication adherence and lifestyle modifications, are essential, implementation gaps remain due to limited health care access and inadequate patient engagement. Telemedical interventions offer a promising solution to these challenges by facilitating remote monitoring and providing individualized patient management strategies.

**Objective:**

This randomized controlled trial aimed to evaluate the efficacy of a comprehensive web-based telemedical interventional management system in reducing major adverse cardiac and cerebrovascular events (MACCE) and enhancing secondary prevention outcomes among patients with CHD following PCI, compared to usual care alone.

**Methods:**

We conducted a single-center, open-label, randomized controlled trial at a tertiary hospital in China. A total of 2086 patients with post-PCI CHD were randomly assigned in a 1:1 ratio to receive either telemedical management combined with usual care (intervention group; n=1040) or usual care alone (control group; n=1046). The control group received follow-up phone calls from health care providers at 1, 3, 6, and 12 months after discharge. In contrast, the remote patient management group benefited from multicomponent interventions delivered through a telemedicine platform, alongside usual care. This platform provided personalized health education, medication reminders, vital sign monitoring, and artificial intelligence-assisted consultations. The primary outcome was the composite incidence of MACCE, including cardiac death, myocardial infarction, stroke, or target vessel revascularization, at one year. Secondary outcomes included bleeding events, lifestyle changes, blood pressure control, and medication adherence.

**Results:**

At the one-year follow-up, the intervention group demonstrated a significant reduction in MACCE compared to the control group (36/1040, 3.5% vs 55/1046, 5.3%, *P=*.04). This was primarily attributed to lower rates of cardiac death (10/1040, 1.0% vs 24/1046, 2.3%, *P=*.02) and myocardial infarction (8/1040, 0.8% vs 19/1046, 1.8%, *P=*.03). Additionally, bleeding events classified as BARC 3‐5 were less frequent in the intervention group (6/1040, 0.6% vs 16/1046, 1.6%, *P=*.03). The intervention group also exhibited improved control over systolic blood pressure (mean 117.74, SD 13.80 mmHg vs mean 121.46, SD 16.85 mmHg, *P=*.002) and diastolic blood pressure (mean 73.60, SD 10.18 mmHg vs mean 75.72, SD 10.45 mmHg, *P=*.02), along with higher medication adherence to aspirin (896/1021, 87.8% vs 858/1017, 84.4%, *P=*.03) and angiotensin-converting enzyme inhibitors, angiotensin receptor blockers, or angiotensin receptor-neprilysin inhibitors (489/1021, 47.9% vs 442/1017, 43.5%, *P=*.045). Furthermore, there was a notable reduction in alcohol consumption among participants in the intervention group (119/1021, 11.7% vs 168/1017, 16.5%, *P*=.002), alongside a trend towards decreased smoking rates (114/1021, 11.2% vs 142/1017, 14.0%, *P*=.06).

**Conclusions:**

Telemedical interventional management significantly enhanced clinical outcomes by reducing MACCE and improving risk factor control among patients with CHD who underwent PCI. These findings underscore the potential of telemedicine to bolster secondary prevention efforts and long-term care strategies. Further multicenter studies are necessary to validate these results and optimize telemedicine frameworks for broader implementation.

## Introduction

Secondary prevention of coronary heart disease (CHD) aims to prevent recurrent coronary events following a clinical diagnosis [1,2]. High adherence to secondary prevention interventions, particularly aggressive lifestyle modifications and evidence-based pharmacotherapy, can substantially reduce the incidence of recurrent coronary events [3]. However, cardiac rehabilitation and secondary prevention programs, typically conducted during outpatient visits, have been underutilized due to challenges such as low accessibility and limited availability, especially in China [4-6]. Moreover, effective self-management of cardiovascular risk factors by patients is often challenging without structured support. The INTERASPIRE study [7] revealed inadequate implementation of guideline standards for secondary prevention within the first year of post-CHD hospitalization, highlighting geographic disparities. In the Chinese cohort, only 36.1% achieved the blood pressure target (<130/80 mmHg), 46.4% met the LDL-C goal (<1.4 mmol/L), and 46.9% attained glycemic control (HbA1c <7.0%). Among those who were smokers at hospitalization, 60.4% continued smoking at follow-up interviews. Participation in cardiac rehabilitation was notably low, reported by only 2% of patients [7]. Furthermore, the utilization of key classes of evidence-based medications—including antiplatelet or anticoagulant agents, beta-blockers, angiotensin-converting enzyme (ACE) inhibitors or angiotensin II receptor blockers (ARBs), and lipid-lowering drugs—was significantly lower among Chinese patients with CHD than among their counterparts in other participating countries [7].

Given the escalating burden of CHD coupled with an aging population, there is an urgent need for new and effective intervention strategies aimed at enhancing postdischarge management in patients with CHD [8]. Despite advancements in percutaneous coronary intervention (PCI), the long-term risk of subsequent cardiovascular events remains elevated among individuals with CHD [9], primarily due to poor adherence to secondary prevention strategies and restricted access to follow-up care. Consequently, innovative and effective interventions are critically required to address these challenges in the future.

Emerging technologies, such as web-based remote patient management systems, have demonstrated efficacy in promoting better self-management by providing health education and facilitating interactions between patients and health care providers [10]. Over the past decade, cardiac rehabilitation and secondary prevention programs utilizing mobile health (mHealth) solutions have been developed, yielding promising results in areas such as medication adherence, smoking cessation, weight loss, physical activity engagement, and health education [5,11-18]. Furthermore, these programs have shown potential in enhancing the risk factor profiles of patients with CHD, while possibly reducing their mortality rates over time [19]. However, their impact on clinical outcomes, specifically recurrent cardiovascular events and bleeding incidents, has yet to be fully elucidated.

Therefore, we established a multicomponent medical intervention framework on a web-based platform for patients with CHD based on standardized management guidelines. This model has the potential to enhance the existing care workforce by integrating patients’ cardiovascular health information and facilitating digital communication between healthcare professionals and patients. We hypothesized that these interventions would be more effective in improving clinical outcomes than usual care alone in patients with CHD. To test this hypothesis, we conducted a single-center, open-label, randomized trial.

## Methods

### Study Design and Participants

This study was conducted at The First Affiliated Hospital of the University of Science and Technology of China (USTC), a prominent tertiary hospital located in Anhui Province, China. Patients were included if they met all the following inclusion criteria: (1) aged between 18 and 79 years (inclusive); (2) presented with clinical manifestations consistent with CHD and successfully underwent PCI; (3) signed an informed consent form, demonstrated the ability to complete follow-up visits, and arrived at the hospital independently; and (4) possessed proficiency in using smartphones and WeChat, a widely used social interaction application in China.

Patients who met any of the following exclusion criteria were not eligible for participation: (1) inability to use the smart management system; (2) New York Heart Association (NYHA) functional class IV; (3) experiencing unstable conditions or complications post-PCI during their current hospitalization; (4) having cardiovascular diseases such as stroke, heart failure, or severe arrhythmias (eg, high-degree atrioventricular block or ventricular tachycardia) within the past three months; (5) having chronic renal insufficiency (creatinine >265 µmol/L); (6) women who are pregnant or breastfeeding; (7) presenting other contraindications for trial participation including thyroid disease requiring medication, acute infectious diseases, psychiatric disorders, psychological conditions, or a history of tumor disease within five years; and (8) having a spouse already enrolled in this study.

All participants were required to possess at least one personal smartphone equipped with an active WeChat account, a widely used social interaction platform in China, and to demonstrate adequate proficiency in the Chinese language to communicate effectively with healthcare providers via WeChat.

For all eligible patients, recruitment and written informed consent were obtained by clinical research coordinators prior to hospital discharge. Subsequently, an independent research coordinator, who was not involved in recruitment, performed the randomization. Using a centralized, computer-generated schedule created with SPSS (version 29.0; IBM Corp), participants were allocated in a 1:1 ratio to either the intervention group (which received remote patient management plus usual care) or the control group (which received usual care only). This process ensured allocation concealment, meaning the recruiting investigators were blinded to the group assignment.

### Ethical Considerations

This study was approved by the Ethics Committee of The First Affiliated Hospital of the University of Science and Technology of China (Approval No. 2022-ky233) and registered with the Chinese Clinical Trial Registry (ChiCTR2200065344). All participants provided written informed consent prior to enrollment. All participants were informed of their right to withdraw from the study at any point. No financial compensation was offered to avoid coercion; telemedicine services were provided free of charge as part of the research intervention.

All data collection, storage, and transmission procedures were conducted in strict compliance with the key data protection and privacy laws of the People's Republic of China [20-23]. These relevant laws and regulations mandate informed consent, data minimization, encryption, and access control to safeguard participants’ personal and medical information. Only authorized study personnel had access to deidentified data, and no third-party commercial use was permitted.

### Treatment

The treatment strategy and stenting techniques used during hospitalization were determined at the discretion of the attending physician. Following PCI, lifelong aspirin therapy was recommended, and P2Y12 antagonists (either clopidogrel or ticagrelor) were prescribed for a duration of one year. Long-term statin therapy was also recommended. The decision regarding the use of other medications, such as beta-blockers, angiotensin-converting enzyme inhibitors (ACEIs), angiotensin II receptor blockers (ARBs), or angiotensin receptor neprilysin inhibitors (ARNIs), was made by the physicians responsible for patient care.

### Usual Care

Before hospital discharge, the standard care provided by ward nurses included guidance on lifestyle modifications and self-management strategies, as recommended for patients with CHD by contemporary clinical guidelines [24-26]. At 1, 3, 6, and 12 months post-discharge, all participants received follow-up calls from health care providers. During these telephone interviews, self-reported information regarding clinical events, symptoms, management of cardiovascular disease risk factors, and prescribed medications was collected. Patients assessed to be at high risk were advised to seek further consultation at their local hospital’s outpatient clinic during follow-up calls. All patients received standard care throughout the study.

### Telemedical Interventional Management

**Figure 1.**
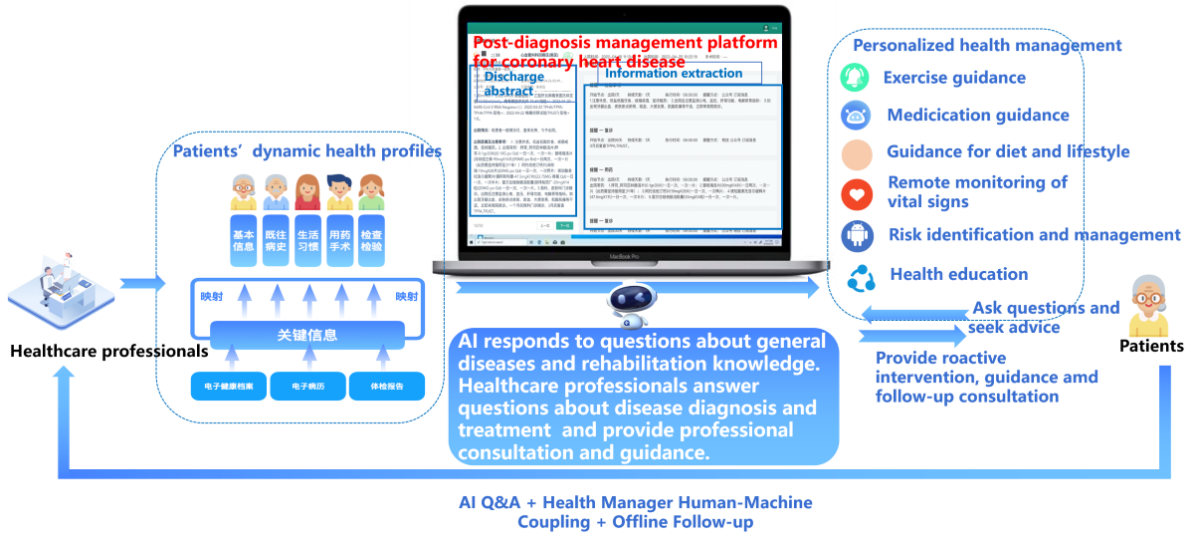
Workflow of the telemedicine platform for post-discharge management. AI: artificial intelligence.

Telemedical interventional management was facilitated through our web-based management platform developed in collaboration with the hospital and Xunfei Healthcare Technology Co. Ltd. (Xunfei Healthcare) to enable remote interventions. The platform (Figure 1) provided health care providers with access to comprehensive cardiovascular health information for patients, including hospitalization records, medications, and laboratory results from their current admission. This access allowed for more informed decision-making regarding diagnosis and ongoing management.

**Figure 2.**
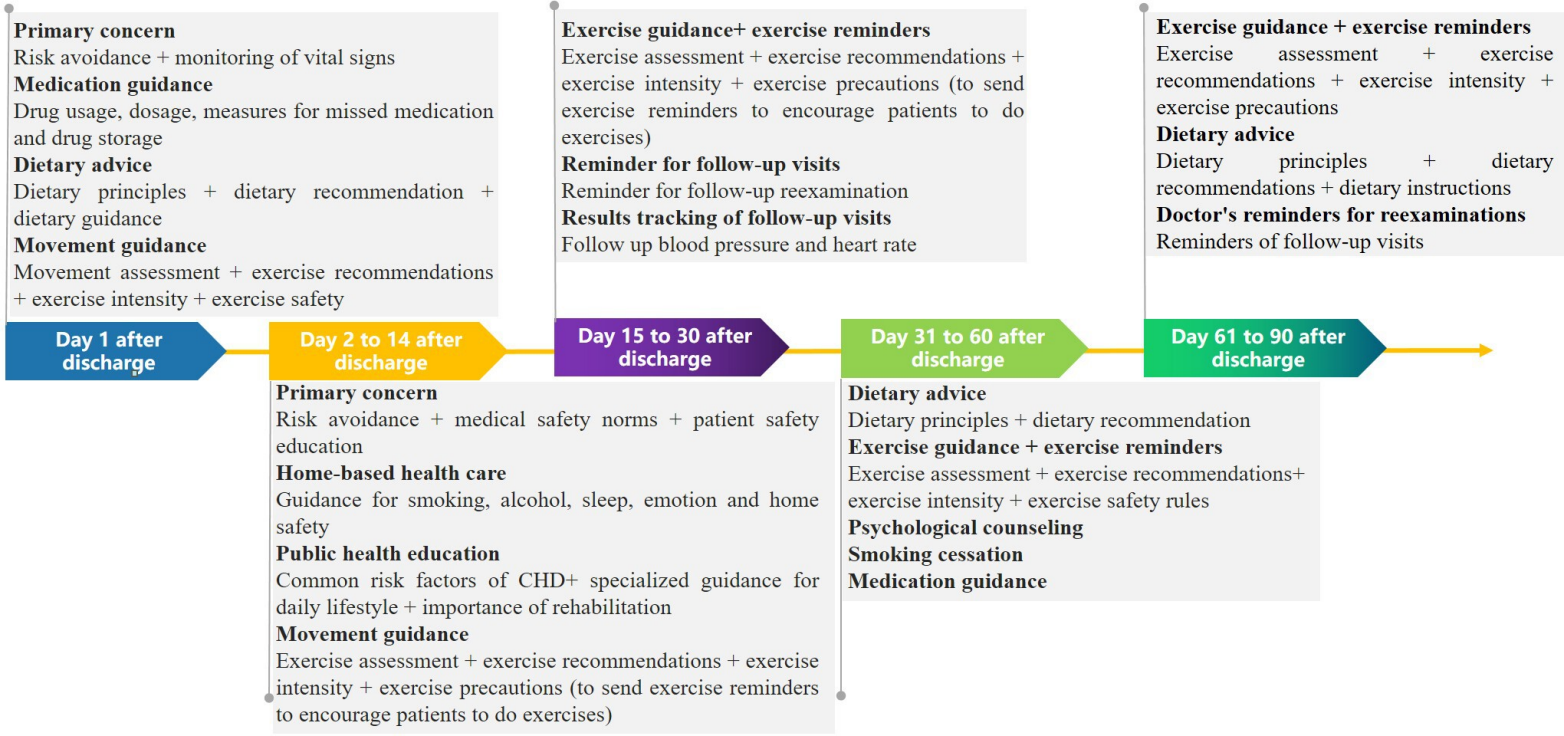
90-day post-discharge care pathway for coronary heart disease patients undergoing percutaneous coronary intervention.

Additionally, the platform offered educational resources and tools aimed at empowering patients to self-manage their conditions while regularly overseeing chronic disease follow-up, supervision, medication reminders, and consultations for most patients. This functionality enabled individuals to engage with personalized self-learning health management materials tailored to their specific conditions and receive precise recommendations for lifestyle modifications (Figure 2 and Multimedia Appendix 1).

The digital platform also dispatched automated follow-up questionnaires (as illustrated in Multimedia Appendix 2), designed to monitor patients’ adherence to medication regimens, lifestyle choices, and clinical events, which are particularly critical for poststent patients. For older adults or less technologically adept individuals (eg, those experiencing difficulties using WeChat) who did not respond to these follow-up questionnaires, data collection was supported through Intelligent Language Tracking and Follow-Up.

Patients in the intervention group were encouraged to seek guidance on lifestyle modifications, symptom management, medications, vital sign monitoring, including heart rate, blood pressure, weight changes, mood assessments, and physical activity tracking (MultimediaAppendices 3  4). Artificial intelligence (AI) capabilities were available on the platform to address inquiries related to general diseases and rehabilitation knowledge (Multimedia Appendix 5); however, questions specifically concerning cardiovascular disease diagnosis and evaluation were addressed by qualified health care professionals. Only patients randomized to the intervention group received telemedical intervention management services.

### Personnel Roles in Telemedical Management

The telemedical intervention was performed by a multidisciplinary team with clearly defined roles. The responsibilities of the personnel involved in delivering the telemedical intervention were as follows:

- Health Managers

- Acted as primary contacts for patients
- Monitored patient data
- Triaged inquiries
- Addressed questions regarding vital signs, medications, physical discomfort, and general health knowledge
- Escalated complex cases to clinicians
- Coordinated follow-ups, reminders, and educational materials

- Nurses

- Provided expert nursing guidance
- Responded to clinical nursing inquiries
- Reinforced patient education, and supported health managers

- Doctors (physicians)

- Offered physician consultations
- Addressed complex clinical queries, including diagnosis, symptom evaluation, test results, and medical adjustments
- Made clinical recommendations
- Reviewed escalated cases
- Remained available for urgent consultations during daytime hours
- Ensured the appropriateness of AI-driven clinical decisions

### Outcome

The primary outcome was a composite of major adverse cardiac or cerebral events (MACCE), a composite of cardiac death, myocardial infarction (MI), target vessel revascularization (TVR), or stroke, assessed during the 1-year follow-up after randomization.

Secondary outcomes included (1) all-cause death, (2) non-cardiac death, (3) stent thrombosis, (4) the first unplanned heart failure or angina hospitalization, (5) bleeding as defined by the Bleeding Academic Research Consortium (BARC) definition [27], (6) smoking and drinking status, (7) office blood pressure, and (8) adherence to cardioprotective medication. All secondary outcomes were assessed at the one-year follow-up after randomization.

Cardiac death was defined as any death with a clear relationship to underlying coronary heart disease (including sudden, unobserved, and unexpected deaths). Noncardiac death was defined as any death in which the primary cause was clearly related to another condition (eg, trauma, cancer, or suicide). MI was defined according to the Fourth Universal Definition of Myocardial Infarction [28]. TVR was defined as the performance of either second PCI or coronary artery bypass grafting (CABG) due to restenosis at the target lesion or any segment of the same major coronary artery. Stroke was defined as a new focal neurological deficit lasting >24 hours, confirmed by neuroimaging and adjudicated by neurologists. Both ischemic and hemorrhagic strokes were considered endpoints, and adjudication was required to differentiate between the two types. Stent thrombosis was defined for additional analyses based on the modified Academic Research Consortium (ARC) definitions [29]. Only definite stent thrombosis was considered an endpoint in this study.

All MACCE and bleeding events were reviewed by an independent Clinical Endpoint Committee (CEC) comprising cardiologists, neurologists, and interventionalists blinded to treatment allocation.

### Estimated Sample Size

The sample size was determined based on a comparative analysis of the incidence rates of cardiovascular events following one year of treatment in prior studies [30,31]. We postulated that the rate of MACCE in the usual care group would be 6.5% at one year, with an anticipated reduction of this rate by 40% due to remote patient management. Assuming a two-sided alpha level of .05 and a statistical power of 80%, along with a 1:1 allocation ratio between the usual care and remote management groups, we calculated that the required sample size would be 1044 participants per group. After factoring in an estimated attrition rate of 5%, the final sample size was established as 1100 cases per group, resulting in a total study population of 2200 participants.

### Statistical Analysis

Primary analysis was performed on the full analysis set (FAS) using an intention-to-treat (ITT) approach. It only included those subjects with data at baseline and set one year as the time span to analyze changes in blood pressure, medication, and lifestyle of these participants at the one-year follow-up visit. SPSS software (version 29.0; IBM Corp) was used for statistical analysis. Categorical variables were described as the number of cases and percentages, and continuous variables were described as the mean and SD or median and IQR. The *χ*^2^ test or Fisher exact test were used to compare the count data between groups, and the *t*-test or Mann-Whitney *U*-test of nonparametric statistics was used to compare the groups of continuous variables. Kaplan-Meier (K-M) curves were used to estimate the incidence of endpoint events during the follow-up period in both groups. All tests were two-sided, and differences were considered statistically significant at *P*<.05.

## Results

**Figure 3.**
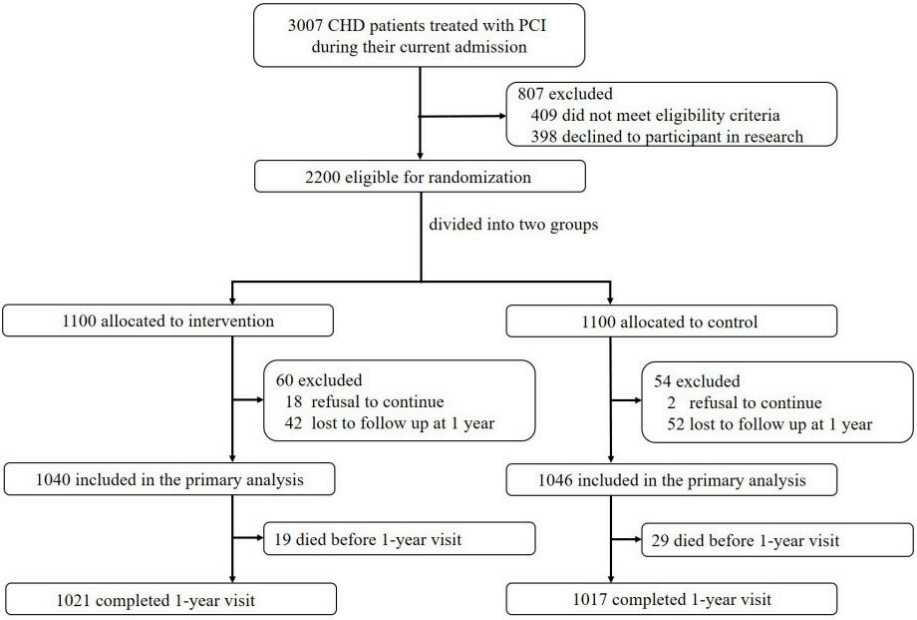
Participant flow diagram. CHD: coronary heart disease; PCI: percutaneous coronary intervention.

Between November 2022 and June 2023, 2086 inpatients diagnosed with CHD who underwent PCI were enrolled in the study (Figure 3). The participants were randomly assigned to two groups: the intervention group, which received remote patient management in addition to usual care (n=1040), and the control group, which received only usual care (n=1046). The baseline clinical and laboratory characteristics as well as the use of cardiovascular medications were comparable between the two groups (Tables1  2).

**Table 1.** Basic clinical and medication characteristics.

Variable	Remote patient management (n=1040)	Usual care (n=1046）	*P* value
Demographics			
Age(y), mean (SD)	62.24 (11.09)	62.00 (10.99)	.61
Sex (Male), n (%)	742 (71.3)	778 (74.5)	.12
Marital status, n (%)			.51
Married	872 (83.8)	888 (84.9)	
Divorced/Widowed/Singlehood	168 (16.2)	158 (15.1)	
Monthly household income, ¥, n (%)			.16
≤5000	467 (44.9)	506 (48.4)	
5000 to <10,000	462 (44.4)	449 (42.9)	
≥10,000	111 (10.7)	91 (8.7)	
Education, n (%)			.78
Junior high school or less	635 (61.1)	645 (61.7)	
High school or higher	405 (38.9)	401 (38.3)	
Clinical data			
Heart rate, mean (SD)	80.90 (16.97)	79.59 (16.89)	.40
Blood pressure (mmHg), mean (SD)			
Systolic	129.90 (19.59)	129.11 (20.85)	.40
Diastolic	79.88 (12.31)	79.79 (12.89)	.93
Distribution of BP, n (%)			
Proportion of patients with BP＞140/90 mmHg	356 (34.2)	355 (33.9)	.89
Proportion of patients with BP＞130/80 mmHg	634 (61.0)	612 (58.5)	.25
BMI (kg/m^2^), mean (SD)	24.82 (3.44)	24.64 (3.46)	.32
Hemoglobin A1c (%), mean (SD)	6.56 (1.38)	6.77 (1.58)	.17
Blood lipids (mmol/L), mean (SD)			
Total cholesterol	4.26 (1.71)	4.27 (1.28)	.90
Triglycerides	1.70 (1.08)	1.76 (1.41)	.40
LDL-C^a^(mmol/L)	2.34 (0.96)	2.39 (0.87)	.28
Hemoglobin(g/L), mean (SD)	129.99 (18.42)	131.24 (23.30)	.22
eGFR^b^ (ml/min/1.73 m^2^), mean (SD)	103.54 (34.44)	100.91 (35.08)	.21
LVEF^c^ (%), median (IQR)	57.00 (48.00‐66.00)	60.50 (49.00‐67.00)	.15
Coronary heart disease types, n (%)		.210	
STEMI^d^	356 (34.2)	397 (38.0)	
NSTEMI^e^	182 (17.5)	159 (15.2)	
Unstable angina pectoris	431 (41.4)	429 (41.0)	
Stable angina pectoris	71 (6.8)	61 (5.8)	
Comorbid conditions, n (%)			
Hypertension	678 (65.2)	675 (64.5)	.75
Diabetes	301 (28.9)	287 (27.4)	.45
History of stroke	109 (10.5)	102 (9.8)	.58
Anemia^f^	154 (14.8)	152 (14.5)	.86
Lifestyle, n (%)			
Current smoking^g^	316 (30.4)	305 (29.2)	.54
Current drinking^h^	308 (29.6)	290 (27.7)	.34
Discharge medications, n (%)			
Aspirin	1021 (98.2)	1023 (97.8)	.55
P2Y12 antagonists	1020 (98.1)	1026 (98.1)	.99
Statins	962 (92.5)	955 (91.3)	.32
ACEI^i^/ARB^j^/ARNI^k^	688 (66.2)	658 (62.9)	.12
Beta-blockers	764 (73.5)	753 (72.0)	.45

a LDL-C: low-density lipoprotein cholesterol.

b eGFR: estimated glomerular filtration rate. eGFR was calculated from serum creatinine (sCr) concentration using the Modified Chinese Equation for GFR Estimation: eGFR (ml/min/1.73 m2)=175×(sCr)−1.234×(age)−0.179×(0.79 if the patient is female).

c LVEF: left ventricular ejection fraction.

d STEMI: ST-segment elevation myocardial infarction.

e NSTEMI: non-ST-segment elevation myocardial infarction.

f Anemia was defined as hemoglobin <110 g/L for women or hemoglobin <120 g/L for men.

g Current smokers were defined as those who smoked at least one cigarette per day.

h Current drinkers were defined as those who drank alcohol at least twice a week for ≥4 consecutive weeks.

i ACEI: angiotensin-converting enzyme inhibitor.

j ARB: angiotensin II receptor blocker.

k ARNI: angiotensin receptor neprilysin inhibitor.

**Table 2.** Procedural characteristics.

Outcomes	Remote patient management (n=1040)	Usual care (n=1046)	*P* value
IRA^a^, n (%)			.22
Left main	56 (5.4)	48 (4.6)	
Left anterior descending	518 (49.8)	566 (54.1)	
Left circumflex	189 (18.2)	166 (15.9)	
Right	277 (26.6)	266 (25.4)	
Disease extent, n (%)			.11
1-vessel disease	355 (34.1)	335 (32.0)	
2-vessel disease	299 (28.7)	345 (33.0)	
3-vessel disease	386 (37.1)	366 (35.0)	
Average stent number, median (IQR)	2 (1-3)	2 (1-3)	.60
Total stent length (mm), median (IQR)	57 (33-84)	57 (33‐72)	.28

a IRA: Infarction related artery.

Throughout the study, four health managers served as full-time staff members at the telemedical center during daytime hours. Additionally, three registered nurses and three registered doctors were tasked with providing health education and addressing patients’ inquiries throughout their hospitalization and during the online follow-up period.

During the trial, all participants in the intervention group received health materials—either text messages or videos—and follow-up questionnaires were sent to them. Among these patients, 81% (843/1040) reported having read the health messages, while the response rate for the follow-up questionnaire was 68% (708/1040). Furthermore, 46% (479/1040) of the patients made inquiries. The total number of online counseling questions in the remote management group was 1973; notably, AI-generated responses accounted for 39% (770/1973) of these answers. Responses from health managers constituted 53% (1046/1973), whereas those from doctors represented only 8% (157/1973). Specifically, inquiries related to illness and cardiac rehabilitation knowledge comprised 47% (928/1973) of all consultation questions posed, consultations regarding vital signs (such as blood pressure and heart rate) and test reports accounted for 19% (375/1973), medication guidance comprised 14% (276/1973), and queries concerning physical discomfort represented 10% (198/1973).

**Figure 4.**
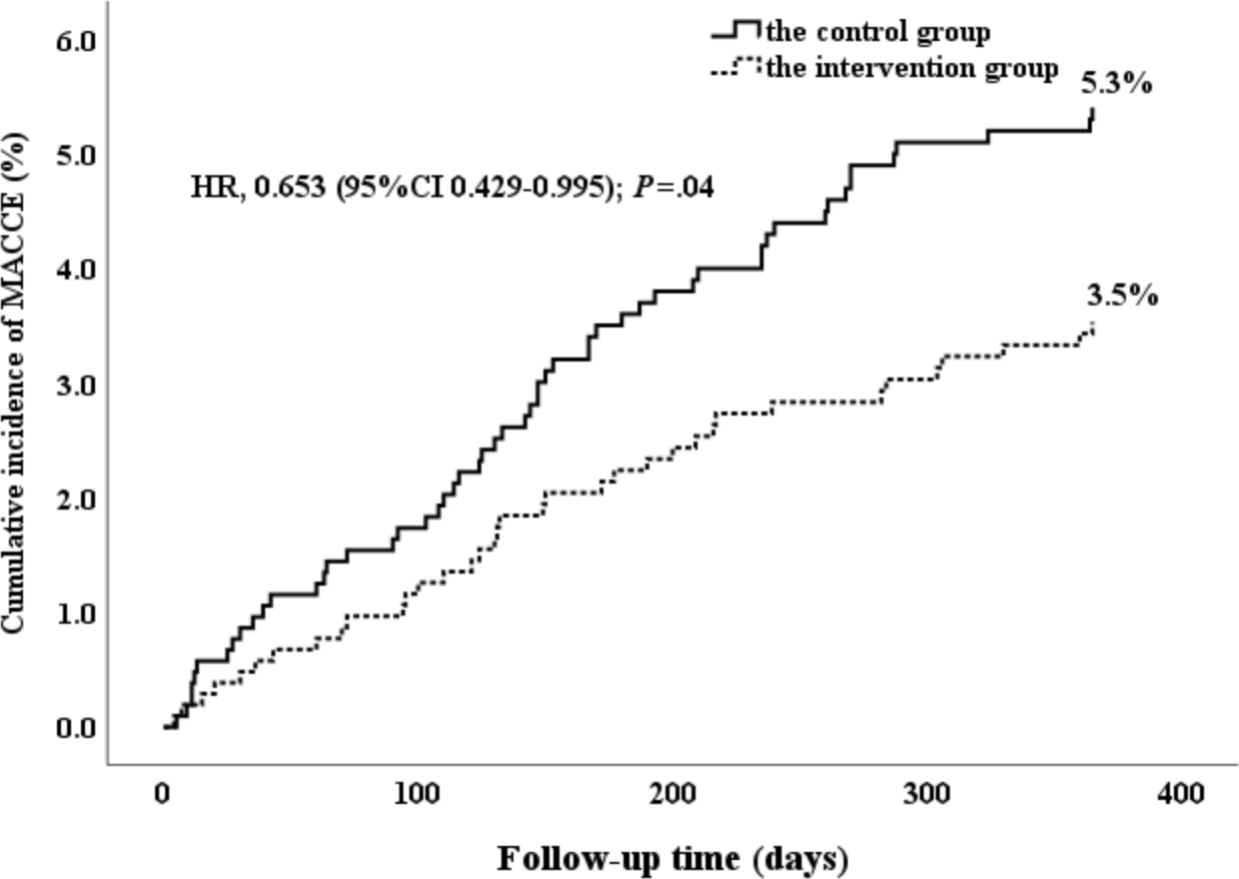
Kaplan-Meier curve for the primary composite endpoint (MACCE). HR: hazard risk; MACCE:major adverse cardiac or cerebral events.

During the one-year follow-up after discharge, 91 MACCE were recorded: 55 MACCE (5.3%) in the usual care group and 36 MACCE (3.5%) in the remote patient management group (Figure 4 and Table 3). The difference between the two groups was statistically significant (*P*=.04). This significance primarily stemmed from a reduction in cardiac death rates (10/1040, 1.0% vs 24/1046, 2.3%, *P*=.02) and myocardial infarction occurrences (8/1040, 0.8% vs 19/1046, 1.8%, *P*=.03) within the remote patient management group compared to the usual care group.

**Table 3.** Adverse events at 1-year follow-up visit.

Outcomes	Remote patient management (n=1040)	Usual care (n=1046)	*P* value
MACCE^a^, n (%)	36 (3.5)	55 (5.3)	.04
Cardiac death	10 (1.0)	24 (2.3)	.02
Myocardial infarction	8 (0.8)	19 (1.8)	.03
Target vessel revascularization	14 (1.4)	17 (1.7)	.59
Stroke	10 (1.0)	14 (1.4)	.41
All-cause mortality, n (%)	19 (1.8)	29 (2.8)	.15
Non-Cardiac death, n (%)	9 (0.9)	5 (0.5)	.29
First unplanned heart failure or angina hospitalization, n (%)	61 (5.9)	66 (6.4)	.65
Stent thrombosis, n (%)	3 (0.3)	5 (0.5)	.48
Any bleeding, n (%)	96 (9.3)	92 (8.9)	.68
BARC^b^1	54 (5.2)	54 (5.2)	.95
BARC^b^2	36 (3.5)	22 (2.1)	.06
BARC^b^3-5	6 (0.6)	16 (1.6)	.03

a MACCE: major adverse cardiac and cerebrovascular events.

b BARC: Bleeding Academic Research Consortium.

No significant differences were observed in stroke incidence (10/1040, 1.0% vs 14/1046, 1.4%), target vessel revascularization (TVR) rates (14/1040, 1.4% vs 17/1046, 1.7%), stent thrombosis (3/1040, 0.3% vs 5/1046, 0.5%), or noncardiac death rates (9/1040, 0.9% vs 5/1046, 0.5%) between the groups, with all comparisons yielding *P*>.05.

Furthermore, no significant difference was noted in BARC 1 bleeding events between the groups (54/1040, 5.2% vs 54/1046, 5.2%, *P*=.95). The remote patient management approach exhibited an increasing trend in BARC 2 bleeding events (36/1040, 3.5% vs 22/1046, 2.1%, *P*=.06) but demonstrated a reduction in BARC 3‐5 bleeding events (6/1040, 0.6% vs 16/1046, 1.6%, *P*=.03).

A total of 1021 patients in the remote patient management group and 1017 in the usual care group completed the extended follow-up. During the one-year follow-up period, significant lifestyle changes were observed among the participants (Table 4). The follow-up data indicated that the remote patient management group exhibited notable differences compared to the usual care group regarding alcohol consumption (119/1021, 11.7% vs 168/1017, 16.5%, *P=*.002). Additionally, remote patient management demonstrated a strong trend towards a reduction in smoking rates (114/1021, 11.2% vs 142/1017, 14.0%, *P*=.06).

**Table 4.** Changes in blood pressure, medication, and lifestyle factors among participants at 1-year follow-up visit.

Lifestyle change	Remote patient management (n=1021)	Usual care (n=1017)	*P* value
Current smoking^a^, n (%)	114 (11.2)	142 (14.0)	.06
Current drinking^b^, n (%)	119 (11.7)	168 (16.5)	.002
Blood pressure control achieved			
Blood pressure(mmHg), mean (SD)			
Systolic	117.74 (13.80)	121.46 (16.85)	.002
Diastolic	73.60 (10.18)	75.72 (10.45)	.02
Distribution of BP, n (%)			
Proportion of patients with BP			
>140/90 mmHg	123 (12.0)	188 (18.5)	<.001
Proportion of patients with BP			
>130/80 mmHg	310 (30.4)	442 (43.5)	<.001
Medication adherence^c^, n (%)			
Aspirin	896 (87.8)	858 (84.4)	.03
P2Y12 antagonists	847 (83.0)	812 (79.8)	.07
Statins	810 (79.3)	776 (76.3)	.10
ACEI^d^/ARB^e^/ARNI^f^	489 (47.9)	442 (43.5)	.045
Beta-blockers	533 (52.2)	516 (50.7)	.51

a Current smokers were defined as those who smoked at least one cigarette per day.

b Current drinkers were defined as those who drank alcohol at least twice a week for ≥4 consecutive weeks.

c Adherence was defined as having medication available to take on ≥80% of the days during the follow-up period (ie, Proportion of Days Covered ≥80%). The Proportion of Days Covered (PDC) for each medication was calculated as the days covered by filled prescriptions divided by the follow-up period from discharge to the last interview, unless therapy was discontinued for ≥3 months. Discontinued medications and approximate discontinuation dates were recorded through interviews and clinical records review.

d ACEI: angiotensin-converting enzyme inhibitor.

e ARB: angiotensin II receptor blocker.

f ARNI: angiotensin receptor neprilysin inhibitor.

In addition, there were significant reductions in both systolic (mean 117.74, SD 13.80 vs mean 121.46, SD 16.85 mmHg, *P=*.002) and diastolic blood pressure (mean 73.60, SD 10.18 vs mean 75.72, SD 10.45 mmHg, *P=*.02) in the remote patient management group compared to the usual care group. At the one-year follow-up, the proportion of patients with blood pressure exceeding 140/90 mmHg was 12.0% (123/1021) in the remote patient management group versus 18.5% (188/1017) in the usual care group. Similarly, a lower percentage of patients in the remote patient management group recorded blood pressure readings above 130/80 mmHg at one year (310/1021, 30.4% vs 442/1017, 43.5%). Both between-group differences remained significant (*P*<.001).

Notably, participants in the remote management group demonstrated higher adherence rates to cardioprotective medications, such as aspirin (896/1021, 87.8% vs 858/1017, 84.4%, *P=*.03) and ACEI, ARB, or ARNI (489/1021, 47.9% vs 442/1017, 43.5%, *P=*.045) after one year of treatment. Furthermore, adherence rates for P2Y12 antagonists (847/1021, 83.0% vs 812/1017, 79.8%, *P*=.07) and statins (810/1021, 79.3% vs 776/1017, 76.3%, *P=*.10) also tended to be greater within the remote patient management cohort; however, these differences did not achieve statistical significance. No significant difference was noted in the adherence rates for beta-blockers.

## Discussion

### Key Findings

Remote medical interventional management demonstrated significant benefits in reducing the incidence of cardiac mortality and myocardial infarction over a one-year study period. Additionally, it led to improvements in BARC 3‐5 bleeding events, blood pressure control, medication adherence, smoking cessation, and reduction of heavy drinking among patients with coronary heart disease following percutaneous coronary intervention.

Our multicomponent interventions delivered through a web-based platform can provide real-time medical guidance to patients, thereby enhancing patient engagement and adherence to chronic CHD management strategies. More importantly, the follow-up period in this trial offers substantial evidence regarding the medium- to long-term clinical effects of technology-assisted cardiac rehabilitation and secondary prevention services on large patient populations.

### Significance of Telemedical Interventional Management

Individuals with CHD have a significantly elevated risk of subsequent cardiovascular events, including myocardial infarction, stroke, and cardiovascular mortality [32,33]. Research indicates that the recurrence rate of acute myocardial infarction can reach as high as 2.5% within one year post-discharge, despite advancements in pharmacological treatments and invasive procedures. Notably, nearly one-third of these recurrent events occur within the first 30 days following discharge; moreover, the one-year mortality rate may be as high as 2.8% [34].

Secondary prevention of CHD focuses on preventing recurrent coronary events after clinical diagnosis. High adherence to secondary prevention interventions, particularly active lifestyle modifications and pharmacotherapy, can lead to a substantial reduction in recurrent coronary incidents. International guidelines strongly endorse evidence-based secondary prevention strategies aimed at mitigating these risks through proven pharmacological therapies, optimization of cardiovascular risk factors, cardiac rehabilitation programs, and strict adherence to dietary and physical activity recommendations [2]. Consequently, long-term follow-up management of patients with CHD after discharge is essential [1]. However, poor treatment adherence and low control rates of cardiovascular risk factors remain prevalent in this patient population [35,36 ]. This situation often arises from insufficient engagement with outpatient services due to inadequate coordination, communication barriers, or limited access [5]. Therefore, innovative and scalable strategies, such as telemedicine, are urgently needed to bridge the gap in post-discharge care and improve the implementation of guideline-directed secondary prevention.

### Efficacy of Telemedical Interventional Management

Secondary prevention in CHD has undergone significant advancements with the introduction of telemedical technologies, which provide innovative and flexible approaches to care. A study involving patients who experienced acute myocardial infarction revealed that those who participated in a digital telerehabilitation intervention demonstrated markedly improved self-health management and a 52% reduction in the risk of readmission within 30 days postdischarge [37]. The SMART-CR/SP (Smartphone and social media-based cardiac rehabilitation and secondary prevention in China) study [5] used smartphones to deliver remote cardiac rehabilitation and secondary prevention guidance services for patients following PCI for CHD. The findings indicated that smartphone-based remote cardiac rehabilitation and secondary prevention significantly enhanced exercise capacity, knowledge of cardiovascular disease prevention and control, blood pressure regulation, heart rate management, lipid control, and adherence to secondary prevention medications among patients with coronary artery disease. Similarly, a recent meta-analysis [19] further illustrated that telemedical interventional management effectively reduced both readmission rates and out-of-hospital mortality in individuals experiencing acute coronary syndromes.

This study extends previous observations by demonstrating that web-based remote patient management is a viable and effective enhancement to standard secondary prevention, significantly improving hard cardiovascular outcomes and reducing major bleeding complications. Several mechanisms might underlie these benefits.

First, our data demonstrated promising effects on medication adherence and the modification of risk factors, including changes in blood pressure and cessation of smoking and alcohol consumption, within this large cohort study. Our findings align with the existing literature. Numerous trials have indicated that remote telehealth interventions play a significant role in reducing cardiovascular risk factors and enhancing adherence to lifestyle modifications, such as medication compliance [5,17], smoking cessation [18], and blood pressure management[19,38,39]. Effective management of these risk factors has been shown to decrease the incidence of coronary disease complications, myocardial infarction, and overall mortality [40,41].

Second, remote patient management transcends the basic concept of patient monitoring and encompasses a comprehensive array of interventions pertinent to patient care. This includes patient education, concurrent medication management, assessment of comorbidities, and personalized recommendations [42]. In our study, participants in the intervention group were afforded the opportunity to access educational resources and consult healthcare providers remotely. Health care professionals can deliver customized educational materials, guidance on lifestyle modifications, and self-management strategies through various platforms. These additional components of remote patient management likely played a significant role in contributing to the observed benefits in our trial.

Third, the telemedicine platform can provide a supportive environment for patients to ask questions, seek clarifications, and receive guidance on effectively managing their conditions. In cardiovascular emergencies, telemedicine can facilitate timely consultations and advice for individuals experiencing abnormalities. Our study revealed that remote patient management was associated with an increased risk of BARC 2 bleeding events (bleeding is classified as minor if it falls under BARC 1‐2 and considered major or fatal if it involves BARC 3‐5 [27]. Specifically, BARC 1 bleeding occurs when the patient does not seek treatment, whereas BARC 2 bleeding necessitates intervention or hospital admission [27]. However, patient adherence to bleeding management protocols and antiplatelet treatment strategies may also contribute to preventing the exacerbation of bleeding episodes. Consequently, this has led to a reduction in the incidence of BARC 3‐5 bleeding events. Similarly, online consultation platforms enable health care providers to promptly assess medical risks and offer immediate recommendations to patients with CHD experiencing chest pain (angina), heart failure, or other serious coronary symptoms. These platforms guide patients or caregivers on the appropriate actions to take prior to reaching a healthcare facility. This approach has the potential to enhance outcomes and decrease mortality rates in critical situations [37].

Overall, web-based remote patient management empowers individuals to assume a more proactive role in overseeing their cardiovascular health, while simultaneously enabling health care professionals to provide personalized and timely care.

### Strengths and Limitations

Our study has both strengths and limitations. This study was a relatively large-scale investigation with a cohort of 2086 patients followed for one year. It primarily focused on evaluating the feasibility, acceptability, and impact of remote management in CHD. To our knowledge, this is the first study to assess the effects of remote health management on bleeding outcomes in patients with CHD following PCI. Additionally, we conducted an in-depth analysis of the determinants and patterns of remote health management. This not only has significant clinical implications but also contributes to establishing a framework for the remote management of patients with CHD.

Our study has certain limitations in the following aspects. First, the findings may not be universally applicable across all cultures, as they are specific to the Chinese context; patients from different cultural or geographic backgrounds may exhibit entirely distinct healthcare management practices. Second, data on several indicators relevant to the development of CHD, such as patients’ lipid levels, glycated hemoglobin, and BMI, were not collected during the follow-up consultations. Consequently, we were unable to discuss the changes in these values. Fortunately, previous studies have provided evidence supporting the association between reduced cardiovascular risk and telemedicine interventions [11,12,43]. Third, some data regarding lifestyle changes and medication adherence were based on self-reports from patients, which could have introduced recall bias. Fourth, although we employed a multi-component intervention design, we could not precisely assess the independent contributions of each component or elucidate the mechanisms that effectively capture change. Finally, although our study demonstrated the benefits associated with telemedicine interventions, air pollution factors were excluded from our analysis. Strong evidence indicates that exposure to PM₂.₅ independently increases recurrent cardiovascular risk in patients with CHD [44]. The absence of regional air quality data and individual exposure assessments hindered our ability to evaluate the potential confounding effects on MACCE outcomes.

### Future Directions

Future studies are recommended to further elucidate the efficacy of telemedical interventional management systems by enhancing the following aspects: First, researchers may incorporate objective measures, such as electronic adherence monitoring, pharmacy refill records, and wearable-derived vital signs, to mitigate self-report bias. Second, they should prospectively collect longitudinal data on risk factors (including lipids, HbA1c, hepatic and renal function, BMI, diet quality, physical activity, and household air pollution) to clarify potential mediators. Third, it is advisable to use study designs that isolate component effects—such as factorial or Sequential Multiple Assignment Randomized Trials—which require predefined component and mediation analyses. These analyses would explore deeper associations among various intervention components and identify the factors that play a pivotal role in enhancing patient prognosis. Finally, researchers can assess generalizability and durability through multicenter studies conducted in diverse geographic and cultural contexts. Additionally, they should routinely capture post-12-month platform engagement metrics to inform long-term optimization efforts.

### Conclusion

In this randomized controlled trial, we compared a comprehensive telemedical interventional management system with usual care in patients with CHD. The telemedicine group exhibited a significant reduction in the composite incidence of MACCE, primarily attributed to the lower rates of cardiac death and myocardial infarction at one year. Additionally, improvements were noted in bleeding events (BARC 3‐5), as well as in the control over systolic and diastolic blood pressure, medication adherence (including aspirin and ACEI, ARB, or ARNI), and alcohol consumption. These findings suggest that telemedical interventions can effectively enhance secondary prevention outcomes in patients with CHD following PCI. Future multicenter studies are warranted to validate these results and optimize the telemedicine frameworks for broader clinical implementation.

## Supplementary material

10.2196/63350 Multimedia Appendix 1 A representative screenshot of the telemedicine platform's user interface (WeChat mini-program) as viewed by a patient in the intervention group. It illustrates the timeline and variety of personalized health education materials pushed to the patient after discharge, including questionnaires, health knowledge articles, and guidance on exercise, nutrition, and medication. This staged, automated delivery of targeted educational content was a core component of the intervention.

10.2196/63350 Multimedia Appendix 2 A sample of the digital follow-up questionnaire administered via the telemedicine platform. It assesses post-discharge clinical events (eg, myocardial infarction, stent thrombosis, rehospitalization), bleeding symptoms, physical activity tolerance, smoking and alcohol use, and medication adherence.

10.2196/63350 Multimedia Appendix 3 A representative consultation dialogue on medication adjustment and vital sign monitoring.

10.2196/63350 Multimedia Appendix 4 A representative consultation dialogue on management of adverse drug reactions (bleeding).

10.2196/63350 Multimedia Appendix 5 Example of an AI response to questions about general diseases and rehabilitation knowledge.

10.2196/63350 Checklist 1 CONSORT-eHEALTH Checklist (V1.6.1).

## Abbreviations

ACEI: angiotensin-converting enzyme inhibitor
ARB: angiotensin II receptor blocker
ARNI: angiotensin receptor neprilysin inhibitor
BARC: bleeding academic research consortium
BP: blood pressure
CHD: coronary heart disease
eGFR: estimated glomerular filtration rate
LDL-C: low-density lipoprotein cholesterol
LVEF: left ventricular ejection fraction
MACCE: major adverse cardiovascular and cerebrovascular events
NSTEMI: non-ST-segment elevation myocardial infarction
NYHA: New York Heart Association
PCI: percutaneous coronary intervention
sCr: serum creatinine
STEMI: ST-segment elevation myocardial infarction
