# Supplementary figures and images for: Efficacy of Telemedical Interventional Management in Patients with Coronary Heart Disease Undergoing Percutaneous Coronary Intervention: Randomized Controlled Trial
Source: J Med Internet Res. 2025 Oct 20;27:e63350. doi: 10.2196/63350 (PMC12536921; doi:10.2196/63350)

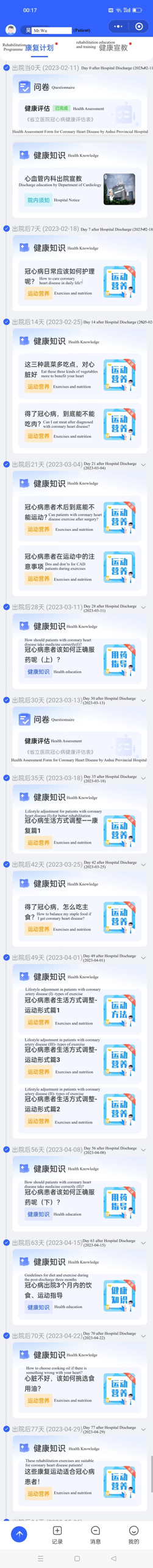

Supplement: Multimedia Appendix 1 [file jmir-v27-e63350-s001.png]

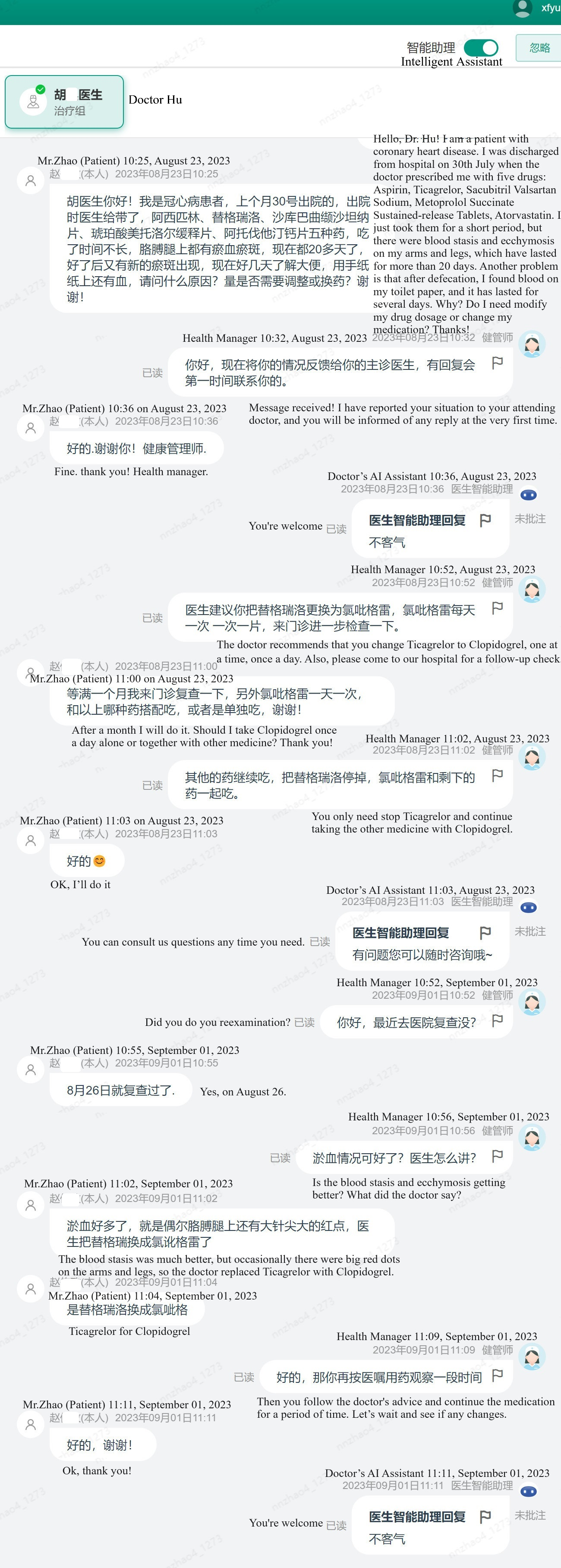

Supplement: Multimedia Appendix 4 [file jmir-v27-e63350-s004.png]

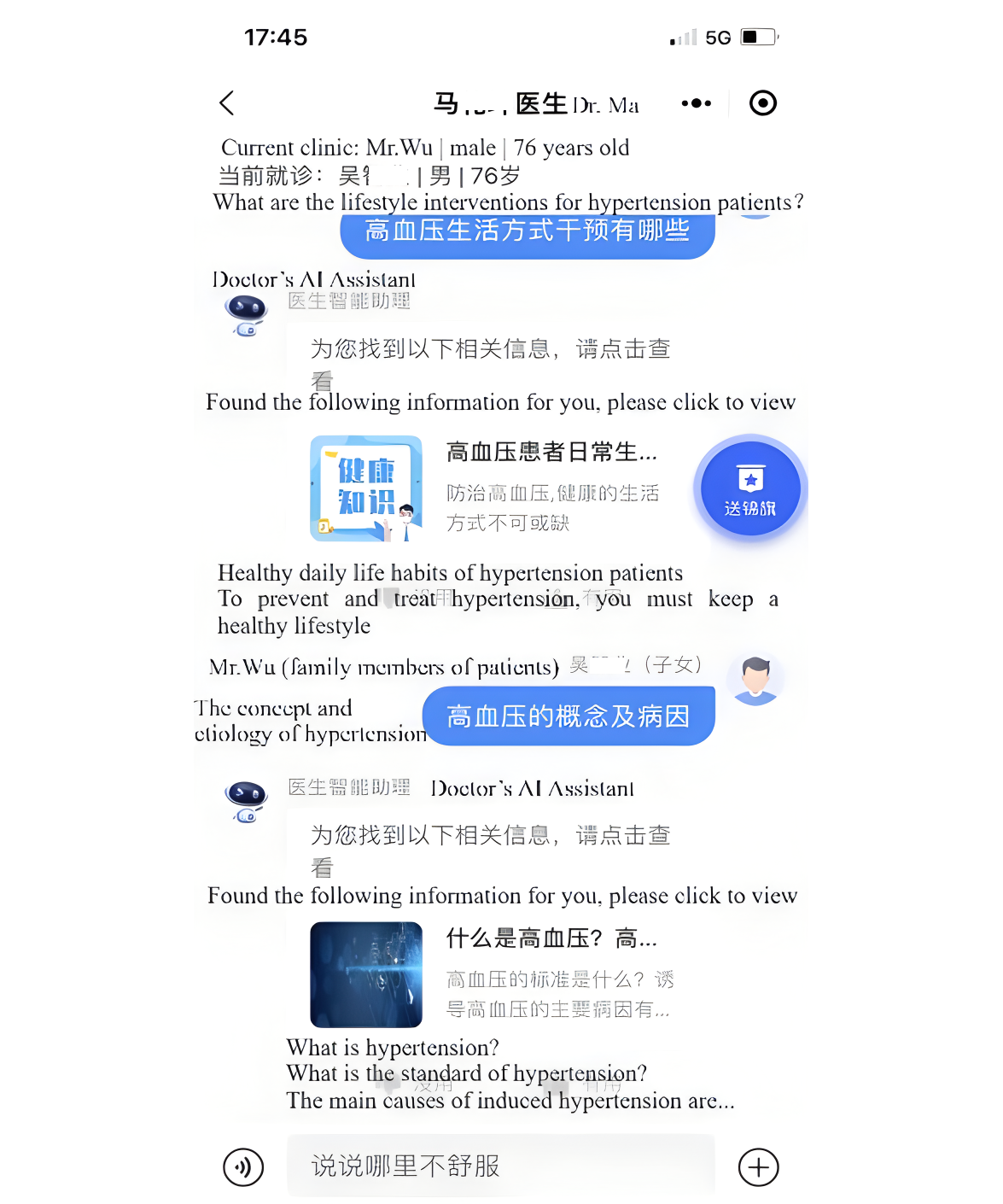

Supplement: Multimedia Appendix 5 [file jmir-v27-e63350-s005.png]
