# Supplementary material for: Efficacy of Telemedical Interventional Management in Patients with Coronary Heart Disease Undergoing Percutaneous Coronary Intervention: Randomized Controlled Trial
Source: J Med Internet Res. 2025 Oct 20;27:e63350. doi: 10.2196/63350 (PMC12536921; doi:10.2196/63350)
Supplement: Multimedia Appendix 2 [file jmir-v27-e63350-s002.pdf]

提交完成

您的问卷已提交，感谢您的参与，祝您身体健康！

咨询医生

有问题问医生，和医生交个朋友

心内科支架患者随访问卷

1.请问您是患者本人吗？

- ◉ A.是
- ○ B.不是

2.请问您/患者自从做完支架手术出院后，是否出现过脑梗死症状？

- ◉ A.有
- ○ B.没有

3.请问具体出现脑梗死的时间（请填写具体年月）

2022年

4.请问您/患者自从做完支架手术出院后，是否出现过术后支架内血栓？

- ◉ A.有
- ○ B.没有

5.请问具体出现术后支架内血栓的时间（请填写具体年月）

2022年

6.请问您/患者自从做完支架手术出院后，是否出现过心肌梗死？

- ◉ A.有
- ○ B.没有

7.请问具体出现心肌梗死的时间（请填写具体年月）

2022年

8.请问您/患者是否在术后支架血管中再次放置支架？

- ◉ A.有
- ○ B.没有

9.请问您/患者支架血管中再次放置支架的时间（请填写具体年月）

2022年

10.请问您/患者自从做完支架手术出院后，是否再次住院？

- ◉ A.有
- ○ B.没有

11.请问您/患者再次住院原因？（多选）

- ○ A.心衰
- ◉ B.心绞痛
- ○ C.脑卒中
- ○ D.高血压
- ○ E.其他原因

12.请问具体再次住院的时间（请填写：原因，住院年月，如有多次请多次填写）

2022年

13.请问您/患者出院后是否出现以下情况？

- ○ A.黑便、尿血、鼻出血、牙龈出血或皮肤瘀斑等
- ○ B.因明显出血症状接受治疗
- ◉ C.无出血

14.请问您/患者出院后是否出现下列情况？

- ○ A.日常活动量不受限制：一般体力活动不引起过度疲劳、心悸、气喘或心绞痛
- ○ B.日常活动量轻微限制。休息时无自觉症状，一般体力活动引起过度疲劳、心悸、气喘或心绞痛。
- ○ C.体力活动明显受限制。休息时无症状，但小于一般体力活动即可引起过度疲劳、心悸、气喘或心绞痛。
- ◉ D.不能从事任何体力活动。休息状态下也出现心悸、气喘或心绞痛症状，体力活动后加重。

15.请问您/患者是否吸烟？（包括卷烟、雪茄、烟斗、水烟等，不包括电子烟和无烟烟草产品）

- ◉ A.目前吸烟
- ○ B.曾经吸烟，已戒烟
- ○ C.从不吸烟

16.在过去的30天，患者有多少天是吸烟的？（请填写数字或不知道）

30

17.请问您/患者每天吸多少根烟（请填写数字或不知道）

1

18.请问您/患者是否饮酒？

- ○ A.目前饮酒
- ○ B.曾经饮酒，已戒酒
- ◉ C.从不饮酒

19.请问患者出院后药物服用情况

- ◉ A.出院药物一直遵循医嘱服药且没有换药
- ○ B.出院后复查调整过药物
- ○ C.出院后未遵循医嘱服药

20.请问患者出院后服用的药物名称（可填写不记得药名、未服药）

阿司匹林

Cardiology Stent Patient Follow-up Questionnaire

Patient Questionnaire

1. Are you the patient yourself?

A. Yes

B. No

Response:

2. Since being discharged after the stent surgery, have you/the patient experienced any symptoms of cerebral infarction?

A. Yes

B. No

Response:

3. If yes, when did the cerebral infarction occur? (Please specify year and month)

Response:

4. Since being discharged after the stent surgery, have you/the patient experienced in-stent thrombosis?

A. Yes

B. No

Response:

5. If yes, when did the in-stent thrombosis occur? (Please specify year and month)

Response:

6. Since being discharged after the stent surgery, have you/the patient experienced myocardial infarction?

A. Yes

B. No

Response:

7. If yes, when did the myocardial infarction occur? (Please specify year and month)

Response:

8. Have you/the patient had another stent placed in the same blood vessel after the initial surgery?

A. Yes

B. No

Response:

9. If yes, when was the additional stent placed? (Please specify year and month)

Response:

10. Since being discharged after the stent surgery, have you/the patient been hospitalized again?

A. Yes

B. No

Response:

11. What was the reason for rehospitalization?

A. Heart disease

B. Angina

C. Stroke

D. Bleeding

E. Other reasons

Response:

12. When did the rehospitalization occur? (Please specify: reason, year and month of hospitalization. If multiple times, list all)

Response:

13. Since discharge, have you/the patient experienced any of the following?

A. Blood in urine, nosebleeds, gum bleeding, or skin bruising

B. Received treatment for significant bleeding symptoms

C. No bleeding

Response:

14. Since discharge, which of the following best describes your/the patient's physical activity tolerance?

A. No limitations on daily activities; ordinary physical activity does not cause fatigue, palpitations, shortness of breath, or angina

B. Slight limitation of daily activities. Comfortable at rest, but ordinary physical activity results in fatigue, palpitations, shortness of breath, or angina

C. Marked limitation of physical activity. Comfortable at rest, but less than ordinary physical activity causes fatigue, palpitations, shortness of breath, or angina

D. Unable to perform any physical activity without discomfort. Symptoms of heart failure or angina present at rest; any physical activity increases discomfort

Response:

15. Do you/does the patient smoke? (Includes cigarettes, cigars, pipes, hookah, etc. Excludes e-cigarettes and smokeless tobacco)

A. Currently smoke

B. Former smoker, have quit

C. Never smoked

Response:

16. In the past 30 days, on how many days did the patient smoke? (Please enter number or "don't know")

Response:

17. How many cigarettes does the patient smoke per day? (Please enter number or "don't know")

Response:

18. Do you/does the patient consume alcohol?

A. Currently consume alcohol

B. Former drinker, have quit

C. Never consumed alcohol

Response:

19. Medication adherence after discharge

A. Consistently followed discharge medication regimen without changes

B. Medications adjusted during follow-up visits after discharge

C. Did not follow the prescribed medication regimen after discharge

Response:

20. What medications has the patient been taking after discharge? (Can write "don't remember medication name" or "not taking medication")

Response:
