# Supplementary material for: Efficacy of Telemedical Interventional Management in Patients with Coronary Heart Disease Undergoing Percutaneous Coronary Intervention: Randomized Controlled Trial
Source: J Med Internet Res. 2025 Oct 20;27:e63350. doi: 10.2196/63350 (PMC12536921; doi:10.2196/63350)
Supplement: Checklist 1 [file jmir-v27-e63350-s006.pdf]

CONSORT-EHEALTH (V 1.6.1) - Submission/Publication Form

The CONSORT-EHEALTH checklist is intended for authors of randomized trials evaluating web-based and Internet-based applications/interventions, including mobile interventions, electronic games (incl multiplayer games), social media, certain telehealth applications, and other interactive and/or networked electronic applications. Some of the items (e.g. all subitems under item 5 - description of the intervention) may also be applicable for other study designs.

The goal of the CONSORT EHEALTH checklist and guideline is to be
a) a guide for reporting for authors of RCTs,
b) to form a basis for appraisal of an ehealth trial (in terms of validity)

CONSORT-EHEALTH items/subitems are MANDATORY reporting items for studies published in the Journal of Medical Internet Research and other journals / scientific societies endorsing the checklist.

Items numbered 1., 2., 3., 4a., 4b etc are original CONSORT or CONSORT-NPT (non-pharmacologic treatment) items.
Items with Roman numerals (i., ii, iii, iv etc.) are CONSORT-EHEALTH extensions/clarifications.

As the CONSORT-EHEALTH checklist is still considered in a formative stage, we would ask that you also RATE ON A SCALE OF 1-5 how important/useful you feel each item is FOR THE PURPOSE OF THE CHECKLIST and reporting guideline (optional).

Mandatory reporting items are marked with a red *.
In the textboxes, either copy & paste the relevant sections from your manuscript into this form - please include any quotes from your manuscript in QUOTATION MARKS,
or answer directly by providing additional information not in the manuscript, or elaborating on why the item was not relevant for this study.

YOUR ANSWERS WILL BE PUBLISHED AS A SUPPLEMENTARY FILE TO YOUR PUBLICATION IN JMIR AND ARE CONSIDERED PART OF YOUR PUBLICATION (IF ACCEPTED).
Please fill in these questions diligently. Information will not be copyedited, so please use proper spelling and grammar, use correct capitalization, and avoid abbreviations.

DO NOT FORGET TO SAVE AS PDF _AND_ CLICK THE SUBMIT BUTTON SO YOUR ANSWERS ARE IN OUR DATABASE !!!

Citation Suggestion (if you append the pdf as Appendix we suggest to cite this paper in the caption):
Eysenbach G, CONSORT-EHEALTH Group
[illegible]

Your response is too large. Try shortening some answers.

[illegible]

URL: http://www.jmir.org/2011/4/e126/
doi: 10.2196/jmir.1923
PMID: 22209829

xiaofanyu22@gmail.com Switch account

Not shared

* Indicates required question

Your name *

First Last

Xiaofan Yu

Primary Affiliation (short), City, Country *

University of Toronto, Toronto, Canada

Department of Cardiology, The First Affiliated I

Your e-mail address *

abc@gmail.com

501183943@qq.com

Title of your manuscript *

Provide the (draft) title of your manuscript.

Efficacy of Telemedical Interventional Management in Coronary Heart Disease Patients Undergoing Percutaneous Coronary Intervention

Your response is too large. Try shortening some answers.

Name of your App/Software/Intervention *

If there is a short and a long/alternate name, write the short name first and add the long name in brackets.

Post-PCI TeleCare Program

Evaluated Version (if any)

e.g. "V1", "Release 2017-03-01", "Version 2.0.27913"

Your answer

Language(s) *

What language is the intervention/app in? If multiple languages are available, separate by comma (e.g. "English, French")

Chinese

URL of your Intervention Website or App

e.g. a direct link to the mobile app on app in appstore (itunes, Google Play), or URL of the website. If the intervention is a DVD or hardware, you can also link to an Amazon page.

Your answer

URL of an image/screenshot (optional)

Your answer

Your response is too large. Try shortening some answers.

Accessibility *

Can an enduser access the intervention presently?

- (selected) access is free and open
- access only for special usergroups, not open
- access is open to everyone, but requires payment/subscription/in-app purchases
- app/intervention no longer accessible
- Other:

Primary Medical Indication/Disease/Condition *

e.g. "Stress", "Diabetes", or define the target group in brackets after the condition, e.g. "Autism (Parents of children with)", "Alzheimers (Informal Caregivers of)"

Coronary Heart Disease after PCI

Primary Outcomes measured in trial *

comma-separated list of primary outcomes reported in the trial

the composite incidence of major adverse car

Secondary/other outcomes

Are there any other outcomes the intervention is expected to affect?

Your answer

Your response is too large. Try shortening some answers.

Recommended "Dose" *

What do the instructions for users say on how often the app should be used?

- ○ Approximately Daily
- ○ Approximately Weekly
- ◉ Approximately Monthly
- ○ Approximately Yearly
- ○ "as needed"
- ○ Other:

Approx. Percentage of Users (starters) still using the app as recommended after 3 months *

- ○ unknown / not evaluated
- ○ 0-10%
- ○ 11-20%
- ○ 21-30%
- ○ 31-40%
- ○ 41-50%
- ○ 51-60%
- ○ 61-70%
- ◉ 71%-80%
- ○ 81-90%
- ○ 91-100%
- ○ Other:

Your response is too large. Try shortening some answers.

Overall, was the app/intervention effective? *

- ○ yes: all primary outcomes were significantly better in intervention group vs control
- ◉ partly: SOME primary outcomes were significantly better in intervention group vs control
- ○ no statistically significant difference between control and intervention
- ○ potentially harmful: control was significantly better than intervention in one or more outcomes
- ○ inconclusive: more research is needed
- ○ Other:

Article Preparation Status/Stage *

At which stage in your article preparation are you currently (at the time you fill in this form)

- ○ not submitted yet - in early draft status
- ○ not submitted yet - in late draft status, just before submission
- ○ submitted to a journal but not reviewed yet
- ○ submitted to a journal and after receiving initial reviewer comments
- ◉ submitted to a journal and accepted, but not published yet
- ○ published
- ○ Other:

Your response is too large. Try shortening some answers.

Journal *

If you already know where you will submit this paper (or if it is already submitted), please provide the journal name (if it is not JMIR, provide the journal name under "other")

- ○ not submitted yet / unclear where I will submit this
- ◉ Journal of Medical Internet Research (JMIR)
- ○ JMIR mHealth and UHealth
- ○ JMIR Serious Games
- ○ JMIR Mental Health
- ○ JMIR Public Health
- ○ JMIR Formative Research
- ○ Other JMIR sister journal
- ○ Other:

Is this a full powered effectiveness trial or a pilot/feasibility trial? *

- ○ Pilot/feasibility
- ◉ Fully powered

Manuscript tracking number *

If this is a JMIR submission, please provide the manuscript tracking number under "other" (The ms tracking number can be found in the submission acknowledgement email, or when you login as author in JMIR. If the paper is already published in JMIR, then the ms tracking number is the four-digit number at the end of the DOI, to be found at the bottom of each published article in JMIR)

- ○ no ms number (yet) / not (yet) submitted to / published in JMIR
- ◉ Other: 63350

Your response is too large. Try shortening some answers.

TITLE AND ABSTRACT

1a) TITLE: Identification as a randomized trial in the title

1a) Does your paper address CONSORT item 1a? *

I.e does the title contain the phrase "Randomized Controlled Trial"? (if not, explain the reason under "other")

- (●) yes
- ( ) Other:

1a-i) Identify the mode of delivery in the title

Identify the mode of delivery. Preferably use "web-based" and/or "mobile" and/or "electronic game" in the title. Avoid ambiguous terms like "online", "virtual", "interactive". Use "Internet-based" only if Intervention includes non-web-based Internet components (e.g. email), use "computer-based" or "electronic" only if offline products are used. Use "virtual" only in the context of "virtual reality" (3-D worlds). Use "online" only in the context of "online support groups". Complement or substitute product names with broader terms for the class of products (such as "mobile" or "smart phone" instead of "iphone"), especially if the application runs on different platforms.

	1	2	3	4	5	
subitem not at all important	( )	( )	(●)	( )	( )	essential

Clear selection

Your response is too large. Try shortening some answers.

Does your paper address subitem 1a-i? *

Copy and paste relevant sections from manuscript title (include quotes in quotation marks "like this" to indicate direct quotes from your manuscript), or elaborate on this item by providing additional information not in the ms, or briefly explain why the item is not applicable/relevant for your study

No, the current manuscript title does not explicitly specify the mode of delivery using the preferred terms (e.g., "web-based," "mobile"). The title uses the broader term "Telemedical," which is an acceptable general descriptor but is considered ambiguous according to the specific guidelines provided. The guidelines recommend avoiding such terms in favor of more precise terminology like "web-based" or "mobile". The manuscript describes a web-based platform accessed via mobile devices in the abstract and methods.The phrases were omitted from the title to keep it concise and reader-friendly.

1a-ii) Non-web-based components or important co-interventions in title

Mention non-web-based components or important co-interventions in title, if any (e.g., “with telephone support”).

	1	2	3	4	5	
subitem not at all important	○	○	○	○	○	essential

Does your paper address subitem 1a-ii?

Copy and paste relevant sections from manuscript title (include quotes in quotation marks "like this" to indicate direct quotes from your manuscript), or elaborate on this item by providing additional information not in the ms, or briefly explain why the item is not applicable/relevant for your study

Your answer

Your response is too large. Try shortening some answers.

1a-iii) Primary condition or target group in the title

Mention primary condition or target group in the title, if any (e.g., “for children with Type I Diabetes”) Example: A Web-based and Mobile Intervention with Telephone Support for Children with Type I Diabetes: Randomized Controlled Trial

	1	2	3	4	5	
subitem not at all important	○	○	○	○	◉	essential

Clear selection

Does your paper address subitem 1a-iii? *

Copy and paste relevant sections from manuscript title (include quotes in quotation marks "like this" to indicate direct quotes from your manuscript), or elaborate on this item by providing additional information not in the ms, or briefly explain why the item is not applicable/relevant for your study

The title explicitly identifies the target group as patients with a primary condition of Coronary Heart Disease (CHD), and further specifies that they are in the important clinical subgroup of those Undergoing Percutaneous Coronary Intervention (PCI). This successfully addresses the requirement to mention the primary condition and target group in the title.

1b) ABSTRACT: Structured summary of trial design, methods, results, and conclusions

NPT extension: Description of experimental treatment, comparator, care providers, centers, and blinding status.

Your response is too large. Try shortening some answers.

1b-i) Key features/functionalities/components of the intervention and comparator in the METHODS section of the ABSTRACT

Mention key features/functionalities/components of the intervention and comparator in the abstract. If possible, also mention theories and principles used for designing the site. Keep in mind the needs of systematic reviewers and indexers by including important synonyms. (Note: Only report in the abstract what the main paper is reporting. If this information is missing from the main body of text, consider adding it)

	1	2	3	4	5	
subitem not at all important	○	○	○	◉	○	essential

Clear selection

Does your paper address subitem 1b-i? *

Copy and paste relevant sections from the manuscript abstract (include quotes in quotation marks "like this" to indicate direct quotes from your manuscript), or elaborate on this item by providing additional information not in the ms, or briefly explain why the item is not applicable/relevant for your study

Intervention Description: The abstract successfully lists key features of the intervention: "personalized health education, medication reminders, vital sign monitoring, and artificial intelligence (AI)-assisted consultations." This is a good, concise summary for systematic reviewers.
Comparator (Usual Care) Description: The description of the comparator is weak. It only states that the control group received "follow-up phone calls... at 1, 3, 6, and 12 months after discharge." The main text clarifies that these calls collected "self-reported information regarding clinical events, symptoms, management of cardiovascular disease risk factors, and prescribed medications".
Theories/Principles: The abstract does not mention any underlying theories or principles used to design the intervention.

Your response is too large. Try shortening some answers.

1b-ii) Level of human involvement in the METHODS section of the ABSTRACT

Clarify the level of human involvement in the abstract, e.g., use phrases like “fully automated” vs. “therapist/nurse/care provider/physician-assisted” (mention number and expertise of providers involved, if any). (Note: Only report in the abstract what the main paper is reporting. If this information is missing from the main body of text, consider adding it)

	1	2	3	4	5	
subitem not at all important	○	○	○	○	○	essential

Does your paper address subitem 1b-ii?

Copy and paste relevant sections from the manuscript abstract (include quotes in quotation marks "like this" to indicate direct quotes from your manuscript), or elaborate on this item by providing additional information not in the ms, or briefly explain why the item is not applicable/relevant for your study

Your answer

1b-iii) Open vs. closed, web-based (self-assessment) vs. face-to-face assessments in the METHODS section of the ABSTRACT

Mention how participants were recruited (online vs. offline), e.g., from an open access website or from a clinic or a closed online user group (closed usergroup trial), and clarify if this was a purely web-based trial, or there were face-to-face components (as part of the intervention or for assessment). Clearly say if outcomes were self-assessed through questionnaires (as common in web-based trials). Note: In traditional offline trials, an open trial (open-label trial) is a type of clinical trial in which both the researchers and participants know which treatment is being administered. To avoid confusion, use “blinded” or “unblinded” to indicated the level of blinding instead of “open”, as “open” in web-based trials usually refers to “open access” (i.e. participants can self-enrol). (Note: Only report in the abstract what the main paper is reporting. If this information is missing from the main body of text, consider adding it)

	1	2	3	4	5	
subitem not at all important	○	○	○	○	○	essential

Your response is too large. Try shortening some answers.

Does your paper address subitem 1b-iii?

Copy and paste relevant sections from the manuscript abstract (include quotes in quotation marks "like this" to indicate direct quotes from your manuscript), or elaborate on this item by providing additional information not in the ms, or briefly explain why the item is not applicable/relevant for your study

Your answer

1b-iv) RESULTS section in abstract must contain use data

Report number of participants enrolled/assessed in each group, the use/uptake of the intervention (e.g., attrition/adherence metrics, use over time, number of logins etc.), in addition to primary/secondary outcomes. (Note: Only report in the abstract what the main paper is reporting. If this information is missing from the main body of text, consider adding it)

	1	2	3	4	5	
subitem not at all important	○	○	○	○	○	essential

Does your paper address subitem 1b-iv?

Copy and paste relevant sections from the manuscript abstract (include quotes in quotation marks "like this" to indicate direct quotes from your manuscript), or elaborate on this item by providing additional information not in the ms, or briefly explain why the item is not applicable/relevant for your study

Your answer

Your response is too large. Try shortening some answers.

1b-v) CONCLUSIONS/DISCUSSION in abstract for negative trials

Conclusions/Discussions in abstract for negative trials: Discuss the primary outcome - if the trial is negative (primary outcome not changed), and the intervention was not used, discuss whether negative results are attributable to lack of uptake and discuss reasons. (Note: Only report in the abstract what the main paper is reporting. If this information is missing from the main body of text, consider adding it)

	1	2	3	4	5	
subitem not at all important	○	○	○	○	○	essential

Does your paper address subitem 1b-v?

Copy and paste relevant sections from the manuscript abstract (include quotes in quotation marks "like this" to indicate direct quotes from your manuscript), or elaborate on this item by providing additional information not in the ms, or briefly explain why the item is not applicable/relevant for your study

Your answer

INTRODUCTION

2a) In INTRODUCTION: Scientific background and explanation of rationale

2a-i) Problem and the type of system/solution

Describe the problem and the type of system/solution that is object of the study: intended as stand-alone intervention vs. incorporated in broader health care program? Intended for a particular patient population? Goals of the intervention, e.g., being more cost-effective to other interventions, replace or complement other solutions? (Note: Details about the intervention are provided in “Methods” under 5)

	1	2	3	4	5	
subitem not at all important	○	○	○	○	◉	essential

Your response is too large. Try shortening some answers.

Does your paper address subitem 2a-i? *

Copy and paste relevant sections from the manuscript (include quotes in quotation marks "like this" to indicate direct quotes from your manuscript), or elaborate on this item by providing additional information not in the ms, or briefly explain why the item is not applicable/relevant for your study

Your response is too large. Try shortening some answers.

Yes, the paper addresses this item in the Background and Introduction sections. The text clearly describes the problem, the target population, the solution's role as a complement to standard care, and its goals.
Here are the relevant direct quotes from the manuscript:

Description of the Problem:

"While secondary prevention strategies, such as medication adherence and lifestyle modifications, are essential, implementation gaps remain due to limited healthcare access and inadequate patient engagement."
"However, cardiac rehabilitation and secondary prevention programs, typically conducted during outpatient visits, have been underutilized due to challenges such as low accessibility and limited availability, especially in China"
"Furthermore, the utilization of key classes of evidence-based medications... was significantly lower among Chinese patients with CHD than among their counterparts in other participating countries"

Type of System/Solution and Target Population:
The system is intended as a complement to a broader healthcare program (usual care) for a specific patient population.

"Telemedical interventions offer a promising solution to these challenges by facilitating remote monitoring and providing individualized patient management strategies."
"This randomized controlled trial aimed to evaluate the efficacy of a comprehensive web-based telemedical interventional management system... among CHD patients following PCI, compared to usual care alone."
"Therefore, innovative and scalable strategies, such as telemedicine, are urgently needed to bridge the gap in post-discharge care and improve the implementation of guideline-directed secondary prevention."

Goal of the Intervention:
The primary goal is to improve clinical outcomes by enhancing the implementation of existing guidelines, not to replace standard care.

"We hypothesized that these interventions would be more effective in improving clinical outcomes than usual care alone in patients with CHD."
"These findings underscore the potential of telemedicine to bolster secondary prevention efforts and long-term care strategies."

In summary, the manuscript defines the problem as an implementation gap in secondary prevention for post-PCI CHD patients. The solution is a telemedicine system intended to complement and enhance usual care within the broader healthcare program for this specific population, with the goal of improving clinical outcomes by supporting guideline adherence.

Your response is too large. Try shortening some answers.

2a-ii) Scientific background, rationale: What is known about the (type of) system

Scientific background, rationale: What is known about the (type of) system that is the object of the study (be sure to discuss the use of similar systems for other conditions/diagnoses, if appropiate), motivation for the study, i.e. what are the reasons for and what is the context for this specific study, from which stakeholder viewpoint is the study performed, potential impact of findings [2]. Briefly justify the choice of the comparator.

	1	2	3	4	5	
subitem not at all important	○	○	○	○	○	essential

Your response is too large. Try shortening some answers.

Does your paper address subitem 2a-ii? *

Copy and paste relevant sections from the manuscript (include quotes in quotation marks "like this" to indicate direct quotes from your manuscript), or elaborate on this item by providing additional information not in the ms, or briefly explain why the item is not applicable/relevant for your study

Yes, the paper comprehensively addresses this item in the Introduction section. It reviews what is known about similar systems, states the motivation and context for the study, implies the stakeholder viewpoint, and provides a clear justification for the choice of comparator (usual care).

Here are the relevant direct quotes from the manuscript, categorized to match the sub-item's requirements:

1.Scientific Background:
"Emerging technologies, such as web-based remote patient management systems, ..., while possibly reducing their mortality rates over time ."

2.Rationale:
"However, their impact on clinical outcomes, specifically recurrent cardiovascular events and bleeding incidents, has yet to be fully elucidated."

3.Context and Stakeholder Viewpoint:
The study is performed from the viewpoint of healthcare providers and clinical researchers seeking to improve patient outcomes within the Chinese healthcare context.

4.Justification for the Choice of Comparator:
The choice of "usual care" as the comparator is justified because it represents the current standard against which the incremental benefit of the telemedicine system must be measured.

5.Potential Impact of Findings:
"These findings underscore the potential of telemedicine to bolster secondary prevention efforts and long-term care strategies."This statement highlights the potential impact on clinical practice and patient management strategies.

2b) In INTRODUCTION: Specific objectives or hypotheses

Your response is too large. Try shortening some answers.

Does your paper address CONSORT subitem 2b? *

Copy and paste relevant sections from the manuscript (include quotes in quotation marks "like this" to indicate direct quotes from your manuscript), or elaborate on this item by providing additional information not in the ms, or briefly explain why the item is not applicable/relevant for your study

Yes, the paper clearly addresses this CONSORT subitem in the final paragraph of the Introduction section. It states both the specific objective of the trial and the research hypothesis.

Direct Quote from the Introduction:

"Objective: This randomized controlled trial aimed to evaluate the efficacy of a comprehensive web-based telemedical interventional management system... usual care alone."

"We hypothesized that these interventions would be more effective in improving clinical outcomes than usual care alone in patients with CHD."

These statements explicitly fulfill the requirement to pre-specify the trial's objective and hypothesis in the introduction.

METHODS

3a) Description of trial design (such as parallel, factorial) including allocation ratio

Your response is too large. Try shortening some answers.

Does your paper address CONSORT subitem 3a? *

Copy and paste relevant sections from the manuscript (include quotes in quotation marks "like this" to indicate direct quotes from your manuscript), or elaborate on this item by providing additional information not in the ms, or briefly explain why the item is not applicable/relevant for your study

Yes, the paper clearly addresses this CONSORT subitem in the Methods section.Here are the relevant direct quotes from the manuscript:

Trial Design:
"We conducted a single-center, open-label, randomized controlled trial..." (Abstract, Methods)

Allocation Ratio:

"A total of 2,086 patients... were randomly assigned in a 1:1 ratio to receive either telemedical management combined with usual care (intervention group; n=1,040) or usual care alone (control group; n=1,046)." (Abstract, Methods)
"Participants were randomized in a 1:1 ratio using a computer-generated randomization schedule..." (Methods section)
The manuscript explicitly states that the trial used a parallel-group design (as it compares two groups simultaneously) with an allocation ratio of 1:1.

3b) Important changes to methods after trial commencement (such as eligibility criteria), with reasons

Does your paper address CONSORT subitem 3b? *

Copy and paste relevant sections from the manuscript (include quotes in quotation marks "like this" to indicate direct quotes from your manuscript), or elaborate on this item by providing additional information not in the ms, or briefly explain why the item is not applicable/relevant for your study

No changes to the trial methods or outcomes occurred after the trial commenced.

Your response is too large. Try shortening some answers.

3b-i) Bug fixes, Downtimes, Content Changes

Bug fixes, Downtimes, Content Changes: ehealth systems are often dynamic systems. A description of changes to methods therefore also includes important changes made on the intervention or comparator during the trial (e.g., major bug fixes or changes in the functionality or content) (5-iii) and other “unexpected events” that may have influenced study design such as staff changes, system failures/downtimes, etc. [2].

	1	2	3	4	5	
subitem not at all important	○	○	○	○	○	essential

Does your paper address subitem 3b-i?

Copy and paste relevant sections from the manuscript (include quotes in quotation marks "like this" to indicate direct quotes from your manuscript), or elaborate on this item by providing additional information not in the ms, or briefly explain why the item is not applicable/relevant for your study

Your answer

4a) Eligibility criteria for participants

Your response is too large. Try shortening some answers.

Does your paper address CONSORT subitem 4a? *

Copy and paste relevant sections from the manuscript (include quotes in quotation marks "like this" to indicate direct quotes from your manuscript), or elaborate on this item by providing additional information not in the ms, or briefly explain why the item is not applicable/relevant for your study

Yes, the paper clearly addresses this CONSORT subitem in the "Study Design and Participants" section of the Methods.

The manuscript provides detailed lists of both inclusion and exclusion criteria. Here are the relevant direct quotes:

Inclusion Criteria:
"Patients were included if they met all the following inclusion criteria: 1)aged between 18 and 79 years (inclusive) ... 4) possessed proficiency in using smartphones and WeChat, a widely used social interaction application in China."

Exclusion Criteria:
"Patients who met any of the following exclusion criteria were not eligible for participation: 1) inability to utilize the smart management system...8) having a spouse already enrolled in this study."

These sections provide a clear and specific description of the eligibility criteria for participants in this study.

4a-i) Computer / Internet literacy

Computer / Internet literacy is often an implicit “de facto” eligibility criterion - this should be explicitly clarified.

	1	2	3	4	5	
subitem not at all important	O	O	O	O	O	essential

Does your paper address subitem 4a-i?

Copy and paste relevant sections from the manuscript (include quotes in quotation marks "like this" to indicate direct quotes from your manuscript), or elaborate on this item by providing additional information not in the ms, or briefly explain why the item is not applicable/relevant for your study

Your response is too large. Try shortening some answers.

4a-ii) Open vs. closed, web-based vs. face-to-face assessments:

Open vs. closed, web-based vs. face-to-face assessments: Mention how participants were recruited (online vs. offline), e.g., from an open access website or from a clinic, and clarify if this was a purely web-based trial, or there were face-to-face components (as part of the intervention or for assessment), i.e., to what degree got the study team to know the participant. In online-only trials, clarify if participants were quasi-anonymous and whether having multiple identities was possible or whether technical or logistical measures (e.g., cookies, email confirmation, phone calls) were used to detect/prevent these.

	1	2	3	4	5	
subitem not at all important	○	○	○	○	○	essential

Does your paper address subitem 4a-ii? *

Copy and paste relevant sections from the manuscript (include quotes in quotation marks "like this" to indicate direct quotes from your manuscript), or elaborate on this item by providing additional information not in the ms, or briefly explain why the item is not applicable/relevant for your study

Yes, the paper addresses this item, clarifying that recruitment was offline (face-to-face) and that the trial was a hybrid model with both face-to-face and web-based components.

Recruitment Method (Offline/Face-to-face):

"Recruitment was conducted prior to hospital discharge for all patients who met the eligibility criteria." (Methods section)
This clearly indicates that recruitment was an offline, face-to-face process occurring in the hospital setting.

Web-Based/Remote Component (Intervention and Follow-up):
"Telemedical interventional management was facilitated through our web-based management platform..." (Methods section: Telemedical Interventional Management)
The core intervention was delivered remotely after discharge.

Participant Identity Verification (Not Anonymous):
The study team knew the participants well from their hospitalization. The process of obtaining written informed consent and enrolling patients during their inpatient stay precluded quasi-anonymity or the possibility of multiple identities. Participants were fully identified individuals within the hospital's care system.

Your response is too large. Try shortening some answers.

4a-iii) Information giving during recruitment

Information given during recruitment. Specify how participants were briefed for recruitment and in the informed consent procedures (e.g., publish the informed consent documentation as appendix, see also item X26), as this information may have an effect on user self-selection, user expectation and may also bias results.

	1	2	3	4	5	
subitem not at all important	○	○	○	○	○	essential

Does your paper address subitem 4a-iii?

Copy and paste relevant sections from the manuscript (include quotes in quotation marks "like this" to indicate direct quotes from your manuscript), or elaborate on this item by providing additional information not in the ms, or briefly explain why the item is not applicable/relevant for your study

Your answer

4b) Settings and locations where the data were collected

Does your paper address CONSORT subitem 4b? *

Copy and paste relevant sections from the manuscript (include quotes in quotation marks "like this" to indicate direct quotes from your manuscript), or elaborate on this item by providing additional information not in the ms, or briefly explain why the item is not applicable/relevant for your study

Yes, the paper clearly addresses this CONSORT subitem in the "Study Design and Participants" section.

"This study was conducted as a single-center, open-label, randomized controlled trial at The First Affiliated Hospital of the University of Science and Technology of China (USTC), a prominent tertiary hospital located in Anhui Province, China."

This statement explicitly describes the setting (a tertiary hospital) and the specific location (Anhui Province, China) where the trial was conducted and the data were collected.

Your response is too large. Try shortening some answers.

4b-i) Report if outcomes were (self-)assessed through online questionnaires

Clearly report if outcomes were (self-)assessed through online questionnaires (as common in web-based trials) or otherwise.

	1	2	3	4	5	
subitem not at all important	○	○	○	○	○	essential

Does your paper address subitem 4b-i? *

Copy and paste relevant sections from the manuscript (include quotes in quotation marks "like this" to indicate direct quotes from your manuscript), or elaborate on this item by providing additional information not in the ms, or briefly explain why the item is not applicable/relevant for your study

The paper describes the methods of data collection for different outcomes:
1.Lifestyle data and medication adherence for the intervention group were collected via online questionnaires (self-assessed), with phone follow-up for non-responders.
"The digital platform also dispatched automated follow-up questionnaires... and clinical events..." (Methods: Telemedical Interventional Management)

"For elderly or less technologically adept individuals... who did not respond to these follow-up questionnaires, data collection was supported through Intelligent Language Tracking and Follow-Up." (This implies phone calls).

2.The same data for the control group were collected via telephone interviews (verbally self-assessed).

3.Hard clinical outcomes (MACCE, bleeding) were not self-assessed; they were adjudicated by an independent clinical committee based on medical records and reports.

Your response is too large. Try shortening some answers.

4b-ii) Report how institutional affiliations are displayed

Report how institutional affiliations are displayed to potential participants [on ehealth media], as affiliations with prestigious hospitals or universities may affect volunteer rates, use, and reactions with regards to an intervention.(Not a required item – describe only if this may bias results)

	1	2	3	4	5	
subitem not at all important	○	○	○	○	○	essential

Does your paper address subitem 4b-ii?

Copy and paste relevant sections from the manuscript (include quotes in quotation marks "like this" to indicate direct quotes from your manuscript), or elaborate on this item by providing additional information not in the ms, or briefly explain why the item is not applicable/relevant for your study

Your answer

5) The interventions for each group with sufficient details to allow replication, including how and when they were actually administered

5-i) Mention names, credential, affiliations of the developers, sponsors, and owners

Mention names, credential, affiliations of the developers, sponsors, and owners [6] (if authors/evaluators are owners or developer of the software, this needs to be declared in a "Conflict of interest" section or mentioned elsewhere in the manuscript).

	1	2	3	4	5	
subitem not at all important	○	○	○	○	○	essential

Your response is too large. Try shortening some answers.

Does your paper address subitem 5-i?

Copy and paste relevant sections from the manuscript (include quotes in quotation marks "like this" to indicate direct quotes from your manuscript), or elaborate on this item by providing additional information not in the ms, or briefly explain why the item is not applicable/relevant for your study

Your answer

5-ii) Describe the history/development process

Describe the history/development process of the application and previous formative evaluations (e.g., focus groups, usability testing), as these will have an impact on adoption/use rates and help with interpreting results.

	1	2	3	4	5	
subitem not at all important	○	○	○	○	○	essential

Does your paper address subitem 5-ii?

Copy and paste relevant sections from the manuscript (include quotes in quotation marks "like this" to indicate direct quotes from your manuscript), or elaborate on this item by providing additional information not in the ms, or briefly explain why the item is not applicable/relevant for your study

Your answer

5-iii) Revisions and updating

Revisions and updating. Clearly mention the date and/or version number of the application/intervention (and comparator, if applicable) evaluated, or describe whether the intervention underwent major changes during the evaluation process, or whether the development and/or content was “frozen” during the trial. Describe dynamic components such as news feeds or changing content which may have an impact on the replicability of the intervention (for unexpected events see item 3b).

1	2	3	4	5
○	○	○	○	○

Your response is too large. Try shortening some answers.

Does your paper address subitem 5-iii?

Copy and paste relevant sections from the manuscript (include quotes in quotation marks "like this" to indicate direct quotes from your manuscript), or elaborate on this item by providing additional information not in the ms, or briefly explain why the item is not applicable/relevant for your study

Your answer

5-iv) Quality assurance methods

Provide information on quality assurance methods to ensure accuracy and quality of information provided [1], if applicable.

	1	2	3	4	5	
subitem not at all important	○	○	○	○	○	essential

Does your paper address subitem 5-iv?

Copy and paste relevant sections from the manuscript (include quotes in quotation marks "like this" to indicate direct quotes from your manuscript), or elaborate on this item by providing additional information not in the ms, or briefly explain why the item is not applicable/relevant for your study

Your answer

5-v) Ensure replicability by publishing the source code, and/or providing screenshots/screen-capture video, and/or providing flowcharts of the algorithms used

Ensure replicability by publishing the source code, and/or providing screenshots/screen-capture video, and/or providing flowcharts of the algorithms used. Replicability (i.e., other researchers should in principle be able to replicate the study) is a hallmark of scientific reporting.

	1	2	3	4	5	
subitem not at all important	○	○	○	○	○	essential

Your response is too large. Try shortening some answers.

Does your paper address subitem 5-v?

Copy and paste relevant sections from the manuscript (include quotes in quotation marks "like this" to indicate direct quotes from your manuscript), or elaborate on this item by providing additional information not in the ms, or briefly explain why the item is not applicable/relevant for your study

Your answer

5-vi) Digital preservation

Digital preservation: Provide the URL of the application, but as the intervention is likely to change or disappear over the course of the years; also make sure the intervention is archived (Internet Archive, webcitation.org, and/or publishing the source code or screenshots/videos alongside the article). As pages behind login screens cannot be archived, consider creating demo pages which are accessible without login.

	1	2	3	4	5	
subitem not at all important	○	○	○	○	○	essential

Does your paper address subitem 5-vi?

Copy and paste relevant sections from the manuscript (include quotes in quotation marks "like this" to indicate direct quotes from your manuscript), or elaborate on this item by providing additional information not in the ms, or briefly explain why the item is not applicable/relevant for your study

Your answer

Your response is too large. Try shortening some answers.

5-vii) Access

Access: Describe how participants accessed the application, in what setting/context, if they had to pay (or were paid) or not, whether they had to be a member of specific group. If known, describe how participants obtained “access to the platform and Internet” [1]. To ensure access for editors/reviewers/readers, consider to provide a “backdoor” login account or demo mode for reviewers/readers to explore the application (also important for archiving purposes, see vi).

	1	2	3	4	5	
subitem not at all important	○	○	◉	○	○	essential

Clear selection

Does your paper address subitem 5-vii? *

Copy and paste relevant sections from the manuscript (include quotes in quotation marks "like this" to indicate direct quotes from your manuscript), or elaborate on this item by providing additional information not in the ms, or briefly explain why the item is not applicable/relevant for your study

The manuscript provides some details on how participants accessed the intervention and the financial context.

Here is the relevant information from the manuscript:

How Participants Accessed the Application & Requirements:
"All participants were required... with healthcare providers via WeChat."
This describes the necessary hardware (smartphone), software (WeChat app), and membership (WeChat account) required for access. The setting/context is remote, post-discharge management from home.

Cost/Payment:
"No financial compensation was offered to avoid coercion; telemedicine services were provided free of charge as part of the research intervention."

Access Process: It states the platform was accessed "via WeChat"

"Backdoor" Login or Demo Mode: a screenshot appendix have been created for editors/reviewers.

Your response is too large. Try shortening some answers.

5-viii) Mode of delivery, features/functionalities/components of the intervention and comparator, and the theoretical framework

Describe mode of delivery, features/functionalities/components of the intervention and comparator, and the theoretical framework [6] used to design them (instructional strategy [1], behaviour change techniques, persuasive features, etc., see e.g., [7, 8] for terminology). This includes an in-depth description of the content (including where it is coming from and who developed it) [1]," whether [and how] it is tailored to individual circumstances and allows users to track their progress and receive feedback" [6]. This also includes a description of communication delivery channels and – if computer-mediated communication is a component – whether communication was synchronous or asynchronous [6]. It also includes information on presentation strategies [1], including page design principles, average amount of text on pages, presence of hyperlinks to other resources, etc. [1].

	1	2	3	4	5	
subitem not at all important	○	○	○	○	○	essential

Your response is too large. Try shortening some answers.

Does your paper address subitem 5-viii? *

Copy and paste relevant sections from the manuscript (include quotes in quotation marks "like this" to indicate direct quotes from your manuscript), or elaborate on this item by providing additional information not in the ms, or briefly explain why the item is not applicable/relevant for your study

The manuscript provides a good description of the mode of delivery, features/functionalities/components but lacks detail on the theoretical framework.

Mode of Delivery & Platform:
"Telemedical interventional management was ... Xunfei Healthcare Technology Co. Ltd."

Key Features/Functionalities:
"The digital platform also dispatched automated follow-up questionnaires... and clinical events..."

Tailoring and Feedback:
"Patients in the intervention group were encouraged to seek guidance on... and physical activity tracking." (This implies tracking and feedback).

Content Source/Developer:
The platform was developed in collaboration with the hospital and "Xunfei Healthcare Technology Co. Ltd." Educational materials were likely based on "standardized management guidelines."

Communication Channels (Implied):
Access was via smartphone and WeChat. The platform involved "AI-assisted consultations" and inquiries handled by "health managers," "nurses," and "doctors."

Specific behavior change techniques, communication synchronicity, and presentation strategies: see Multimedia Appendices 3 to 5.

The manuscript lacks detail on the theoretical framework

5-ix) Describe use parameters

Describe use parameters (e.g., intended "doses" and optimal timing for use). Clarify what instructions or recommendations were given to the user, e.g., regarding timing, frequency, heaviness of use, if any, or was the intervention used ad libitum.

	1	2	3	4	5	
subitem not at all important	○	○	○	○	○	essential

Your response is too large. Try shortening some answers.

Does your paper address subitem 5-ix?

Copy and paste relevant sections from the manuscript (include quotes in quotation marks "like this" to indicate direct quotes from your manuscript), or elaborate on this item by providing additional information not in the ms, or briefly explain why the item is not applicable/relevant for your study

Your answer

5-x) Clarify the level of human involvement

Clarify the level of human involvement (care providers or health professionals, also technical assistance) in the e-intervention or as co-intervention (detail number and expertise of professionals involved, if any, as well as “type of assistance offered, the timing and frequency of the support, how it is initiated, and the medium by which the assistance is delivered”. It may be necessary to distinguish between the level of human involvement required for the trial, and the level of human involvement required for a routine application outside of a RCT setting (discuss under item 21 – generalizability).

	1	2	3	4	5	
subitem not at all important	○	○	○	○	○	essential

Does your paper address subitem 5-x?

Copy and paste relevant sections from the manuscript (include quotes in quotation marks "like this" to indicate direct quotes from your manuscript), or elaborate on this item by providing additional information not in the ms, or briefly explain why the item is not applicable/relevant for your study

Your answer

Your response is too large. Try shortening some answers.

5-xi) Report any prompts/reminders used

Report any prompts/reminders used: Clarify if there were prompts (letters, emails, phone calls, SMS) to use the application, what triggered them, frequency etc. It may be necessary to distinguish between the level of prompts/reminders required for the trial, and the level of prompts/reminders for a routine application outside of a RCT setting (discuss under item 21 – generalizability).

	1	2	3	4	5	
subitem not at all important	○	○	◉	○	○	essential

Clear selection

Does your paper address subitem 5-xi? *

Copy and paste relevant sections from the manuscript (include quotes in quotation marks "like this" to indicate direct quotes from your manuscript), or elaborate on this item by providing additional information not in the ms, or briefly explain why the item is not applicable/relevant for your study

Yes, the paper addresses this item, primarily describing the reminders and prompts that were part of the intervention itself.

Prompts/Reminders as a Core Intervention Feature:

"This platform provided personalized health education, medication reminders, vital sign monitoring, and artificial intelligence (AI)-assisted consultations." (Abstract, Methods)
This identifies medication reminders as a key functionality.

Automated System Triggers and Follow-up for Non-Response:

"The digital platform also dispatched automated follow-up questionnaires... designed to monitor patients' adherence..."
"For elderly or less technologically adept individuals... who did not respond to these follow-up questionnaires, data collection was supported through Intelligent Language Tracking and Follow-Up." (Methods: Telemedical Interventional Management)
This indicates that automated prompts (questionnaires) were sent, and a secondary prompt system (likely phone calls) was triggered by non-response.

Your response is too large. Try shortening some answers.

5-xii) Describe any co-interventions (incl. training/support)

Describe any co-interventions (incl. training/support): Clearly state any interventions that are provided in addition to the targeted eHealth intervention, as ehealth intervention may not be designed as stand-alone intervention. This includes training sessions and support [1]. It may be necessary to distinguish between the level of training required for the trial, and the level of training for a routine application outside of a RCT setting (discuss under item 21 – generalizability.

	1	2	3	4	5	
subitem not at all important	○	○	○	○	○	essential

Does your paper address subitem 5-xii? *

Copy and paste relevant sections from the manuscript (include quotes in quotation marks "like this" to indicate direct quotes from your manuscript), or elaborate on this item by providing additional information not in the ms, or briefly explain why the item is not applicable/relevant for your study

Yes, the paper clearly addresses this item by stating that the telemedicine intervention was provided in addition to standard care (a co-intervention), and describes the components of that standard care.

Intervention Group Receiving Telemedicine PLUS Usual Care:

"A total of 2,086 patients... were randomly assigned... to receive either telemedical management combined with usual care (intervention group...)" (Abstract, Methods)
This explicitly states that the eHealth intervention was not stand-alone but was a co-intervention with usual care.

Description of Usual Care (The Co-intervention for both groups):

"All patients received standard care throughout the study." (Methods: Usual Care)
"Before hospital discharge, the standard care provided by ward nurses included guidance on lifestyle modifications and self-management strategies, as recommended for patients with CHD by contemporary clinical guidelines..." (Methods: Usual Care)
"At 1, 3, 6, and 12 months post-discharge, all participants received ... collected." (Methods: Usual Care)

6a) Completely defined pre-specified primary and secondary outcome measures, including how and when they were assessed

Your response is too large. Try shortening some answers.

Does your paper address CONSORT subitem 6a? *

Copy and paste relevant sections from the manuscript (include quotes in quotation marks "like this" to indicate direct quotes from your manuscript), or elaborate on this item by providing additional information not in the ms, or briefly explain why the item is not applicable/relevant for your study

The manuscript provides clear definitions for both the primary and secondary outcomes and specifies when they were assessed.

Primary Outcome Definition and Timing:

"The primary outcome was a composite of major adverse cardiac or cerebral events ... assessed during the 1-year follow-up after randomization."

Secondary Outcomes Definition and Timing:

"Secondary outcomes included: (1) all-cause death, ... after randomization."

Detailed Definitions of Outcome Components:
The manuscript further provides operational definitions for the key components of the primary outcome (e.g., definitions of cardiac death, MI, TVR, stroke) and secondary outcomes (e.g., definition of medication adherence), often citing standard clinical guidelines.

6a-i) Online questionnaires: describe if they were validated for online use and apply CHERRIES items to describe how the questionnaires were designed/deployed

If outcomes were obtained through online questionnaires, describe if they were validated for online use and apply CHERRIES items to describe how the questionnaires were designed/deployed [9].

	1	2	3	4	5	
subitem not at all important	○	○	○	○	○	essential

Does your paper address subitem 6a-i?

Copy and paste relevant sections from manuscript text

Your response is too large. Try shortening some answers.

6a-ii) Describe whether and how “use” (including intensity of use/dosage) was defined/measured/monitored

Describe whether and how “use” (including intensity of use/dosage) was defined/measured/monitored (logins, logfile analysis, etc.). Use/adoption metrics are important process outcomes that should be reported in any ehealth trial.

	1	2	3	4	5	
subitem not at all important	○	○	○	○	○	essential

Does your paper address subitem 6a-ii?

Copy and paste relevant sections from manuscript text

Your answer

6a-iii) Describe whether, how, and when qualitative feedback from participants was obtained

Describe whether, how, and when qualitative feedback from participants was obtained (e.g., through emails, feedback forms, interviews, focus groups).

	1	2	3	4	5	
subitem not at all important	○	○	○	○	○	essential

Does your paper address subitem 6a-iii?

Copy and paste relevant sections from manuscript text

Your answer

6b) Any changes to trial outcomes after the trial commenced, with reasons

Your response is too large. Try shortening some answers.

Does your paper address CONSORT subitem 6b? *

Copy and paste relevant sections from the manuscript (include quotes in quotation marks "like this" to indicate direct quotes from your manuscript), or elaborate on this item by providing additional information not in the ms, or briefly explain why the item is not applicable/relevant for your study

No changes were made to the trial outcomes after the trial commenced.

7a) How sample size was determined

NPT: When applicable, details of whether and how the clustering by care provides or centers was addressed

7a-i) Describe whether and how expected attrition was taken into account when calculating the sample size

Describe whether and how expected attrition was taken into account when calculating the sample size.

	1	2	3	4	5	
subitem not at all important	○	○	○	○	○	essential

Does your paper address subitem 7a-i?

Copy and paste relevant sections from manuscript title (include quotes in quotation marks "like this" to indicate direct quotes from your manuscript), or elaborate on this item by providing additional information not in the ms, or briefly explain why the item is not applicable/relevant for your study

Your answer

7b) When applicable, explanation of any interim analyses and stopping guidelines

Your response is too large. Try shortening some answers.

Does your paper address CONSORT subitem 7b? *

Copy and paste relevant sections from the manuscript (include quotes in quotation marks "like this" to indicate direct quotes from your manuscript), or elaborate on this item by providing additional information not in the ms, or briefly explain why the item is not applicable/relevant for your study

No interim analyses were planned or conducted, and no stopping guidelines were implemented.

8a) Method used to generate the random allocation sequence

NPT: When applicable, how care providers were allocated to each trial group

Does your paper address CONSORT subitem 8a? *

Copy and paste relevant sections from the manuscript (include quotes in quotation marks "like this" to indicate direct quotes from your manuscript), or elaborate on this item by providing additional information not in the ms, or briefly explain why the item is not applicable/relevant for your study

Yes, the paper clearly addresses the first part of this subitem.
"Eligible patients were recruited... or the control group (usual care only). "
This statement explicitly describes the method used to generate the random allocation sequence.

Regarding the second part of the subitem ("When applicable, how care providers were allocated to each trial group"):

Explanation: This part is not applicable to the study. The trial was patient-randomized, not cluster-randomized. The intervention was delivered by a central telemedicine team to all participants in the intervention group. There was no allocation of different care providers to different trial groups; the same team served the entire intervention cohort. Therefore, the method of allocating providers does not need to be described.

8b) Type of randomisation; details of any restriction (such as blocking and block size)

Your response is too large. Try shortening some answers.

Does your paper address CONSORT subitem 8b? *

Copy and paste relevant sections from the manuscript (include quotes in quotation marks "like this" to indicate direct quotes from your manuscript), or elaborate on this item by providing additional information not in the ms, or briefly explain why the item is not applicable/relevant for your study

The manuscript mentions the use of a computer-generated randomization schedule.
Direct Quote from the Manuscript:
“Eligible patients were recruited prior to hospital discharge...Participants were allocated in a 1:1 ratio to either the intervention group (remote management plus usual care) or the control group (usual care only)."
“All MACCE and Bleeding events were reviewed by an independent Clinical Endpoint Committee (CEC) comprising cardiologists, neurologists, and interventionalists blinded to treatment allocation.”
This indicates that although the patients and intervention providers were not blinded, the outcome assessors were blinded to the group information, which helps to reduce bias in the determination of the primary objective outcomes.

9) Mechanism used to implement the random allocation sequence (such as sequentially numbered containers), describing any steps taken to conceal the sequence until interventions were assigned

Does your paper address CONSORT subitem 9? *

Copy and paste relevant sections from the manuscript (include quotes in quotation marks "like this" to indicate direct quotes from your manuscript), or elaborate on this item by providing additional information not in the ms, or briefly explain why the item is not applicable/relevant for your study

The manuscript describes how the random allocation sequence was generated
"Participants were randomized in a 1:1 ratio using a computer-generated randomization schedule created with SPSS version 29.0, assigning them to either the remote patient management plus usual care group (remote management group) or the usual care only group (usual care group). Upon obtaining written informed consent and confirming eligibility, an independent research coordinator who was not involved in participant enrollment accessed the system to obtain the group assignment. "

10) Who generated the random allocation sequence, who enrolled participants, and who assigned participants to interventions

Your response is too large. Try shortening some answers.

Does your paper address CONSORT subitem 10? *

Copy and paste relevant sections from the manuscript (include quotes in quotation marks "like this" to indicate direct quotes from your manuscript), or elaborate on this item by providing additional information not in the ms, or briefly explain why the item is not applicable/relevant for your study

The manuscript describes how the random allocation sequence was generated "Participants were randomized in a 1:1 ratio using ...accessed the system to obtain the group assignment. "

11a) If done, who was blinded after assignment to interventions (for example, participants, care providers, those assessing outcomes) and how

NPT: Whether or not administering co-interventions were blinded to group assignment

11a-i) Specify who was blinded, and who wasn't

Specify who was blinded, and who wasn't. Usually, in web-based trials it is not possible to blind the participants [1, 3] (this should be clearly acknowledged), but it may be possible to blind outcome assessors, those doing data analysis or those administering co-interventions (if any).

	1	2	3	4	5	
subitem not at all important	○	○	○	○	○	essential

Your response is too large. Try shortening some answers.

Does your paper address subitem 11a-i? *

Copy and paste relevant sections from the manuscript (include quotes in quotation marks "like this" to indicate direct quotes from your manuscript), or elaborate on this item by providing additional information not in the ms, or briefly explain why the item is not applicable/relevant for your study

The manuscript correctly acknowledges the lack of blinding for participants and intervention providers, which is typical and often unavoidable in behavioral or telemedicine trials. Crucially, it reports the implementation of blinding for the outcome assessors of the primary endpoints, which is a key methodological strength to minimize ascertainment bias.

Who was NOT blinded (Open-label):

"We conducted a single-center, open-label, randomized controlled trial..." (Abstract, Methods)
The term "open-label" explicitly acknowledges that the participants and the personnel delivering the intervention (health managers, nurses, doctors) were not blinded to the group assignment.

Who WAS blinded (Outcome Assessors):

"All MACCE and Bleeding events were reviewed by an independent CEC comprising cardiologists, neurologists, and interventionalists blinded to treatment allocation." (Methods: Outcome)
This clearly states that the outcome assessors (the CEC) were blinded to the group assignment when adjudicating the primary clinical endpoints.

11a-ii) Discuss e.g., whether participants knew which intervention was the “intervention of interest” and which one was the “comparator”

Informed consent procedures (4a-ii) can create biases and certain expectations - discuss e.g., whether participants knew which intervention was the “intervention of interest” and which one was the “comparator”.

	1	2	3	4	5	
subitem not at all important	○	○	○	○	○	essential

Your response is too large. Try shortening some answers.

Does your paper address subitem 11a-ii?

Copy and paste relevant sections from the manuscript (include quotes in quotation marks "like this" to indicate direct quotes from your manuscript), or elaborate on this item by providing additional information not in the ms, or briefly explain why the item is not applicable/relevant for your study

Your answer

11b) If relevant, description of the similarity of interventions

(this item is usually not relevant for ehealth trials as it refers to similarity of a placebo or sham intervention to a active medication/intervention)

Does your paper address CONSORT subitem 11b? *

Copy and paste relevant sections from the manuscript (include quotes in quotation marks "like this" to indicate direct quotes from your manuscript), or elaborate on this item by providing additional information not in the ms, or briefly explain why the item is not applicable/relevant for your study

This item is not applicable or relevant for our study.

Explanation: As correctly noted in the prompt, CONSORT subitem 11b pertains to trials where a placebo or sham intervention is used to mimic the active intervention (e.g., in drug trials or device trials with sham procedures). The purpose is to describe how well the placebo/sham matches the active intervention to ensure blinding.

Our Study Context: Our trial did not use a placebo or sham telemedicine intervention. The control group received "usual care alone", which is a standard, real-world comparator (consisting of pre-discharge education and scheduled follow-up phone calls). The intervention group received "usual care plus" the telemedicine platform. There was no attempt to create a sham telemedicine platform that looked identical but was inactive. Therefore, describing the "similarity of interventions" is not relevant in this context.

The design is a standard superiority trial comparing an enhanced care model to standard care, not a placebo-controlled trial.

12a) Statistical methods used to compare groups for primary and secondary outcomes

NPT: When applicable, details of whether and how the clustering by care providers or

Your response is too large. Try shortening some answers.

Does your paper address CONSORT subitem 12a? *

Copy and paste relevant sections from the manuscript (include quotes in quotation marks "like this" to indicate direct quotes from your manuscript), or elaborate on this item by providing additional information not in the ms, or briefly explain why the item is not applicable/relevant for your study

The manuscript describes the statistical methods in general terms but lacks specificity for different types of outcomes.

The manuscript addresses the basic statistical methods. Here is the relevant direct quote from the manuscript:

"SPSS v29.0 (IBM Corp., Armonk, NY, USA) was used for statistical analysis... All tests were two-sided, and differences were considered statistically significant at P<.05."

The trial was conducted at a single center. While the intervention was delivered by a team of health managers, nurses, and doctors, the randomization was performed at the patient level, not the provider level (it was not a cluster-randomized trial). Therefore, accounting for clustering was not necessary.

12a-i) Imputation techniques to deal with attrition / missing values

Imputation techniques to deal with attrition / missing values: Not all participants will use the intervention/comparator as intended and attrition is typically high in ehealth trials. Specify how participants who did not use the application or dropped out from the trial were treated in the statistical analysis (a complete case analysis is strongly discouraged, and simple imputation techniques such as LOCF may also be problematic [4]).

	1	2	3	4	5	
subitem not at all important	○	○	○	○	○	essential

Your response is too large. Try shortening some answers.

Does your paper address subitem 12a-i? *

Copy and paste relevant sections from the manuscript (include quotes in quotation marks "like this" to indicate direct quotes from your manuscript), or elaborate on this item by providing additional information not in the ms, or briefly explain why the item is not applicable/relevant for your study

The manuscript states that a primary analysis was performed on the full analysis set (FAS) using an intention-to-treat (ITT) approach.

"Primary analysis was performed on the full analysis set (FAS) using an intention-to-treat (ITT) approach. It only included those subjects with data at baseline and set one year as the time span to analyze changes in blood pressure, medication, and lifestyle of these participants at the one-year follow-up visit."

12b) Methods for additional analyses, such as subgroup analyses and adjusted analyses

Does your paper address CONSORT subitem 12b? *

Copy and paste relevant sections from the manuscript (include quotes in quotation marks "like this" to indicate direct quotes from your manuscript), or elaborate on this item by providing additional information not in the ms, or briefly explain why the item is not applicable/relevant for your study

The manuscript describes the primary statistical methods for comparing groups but does not mention or provide details on subgroup analyses and adjusted analyses.The results of subgroup analyses will be presented in a subsequent manuscript focusing on predictors of response to the telemedical intervention.

X26) REB/IRB Approval and Ethical Considerations [recommended as subheading under "Methods"] (not a CONSORT item)

X26-i) Comment on ethics committee approval

1 2 3 4 5

Your response is too large. Try shortening some answers.

Does your paper address subitem X26-i?

Copy and paste relevant sections from the manuscript (include quotes in quotation marks "like this" to indicate direct quotes from your manuscript), or elaborate on this item by providing additional information not in the ms, or briefly explain why the item is not applicable/relevant for your study

Your answer

x26-ii) Outline informed consent procedures

Outline informed consent procedures e.g., if consent was obtained offline or online (how? Checkbox, etc.?), and what information was provided (see 4a-ii). See [6] for some items to be included in informed consent documents.

	1	2	3	4	5	
subitem not at all important	○	○	○	○	○	essential

Does your paper address subitem X26-ii?

Copy and paste relevant sections from the manuscript (include quotes in quotation marks "like this" to indicate direct quotes from your manuscript), or elaborate on this item by providing additional information not in the ms, or briefly explain why the item is not applicable/relevant for your study

Your answer

X26-iii) Safety and security procedures

Safety and security procedures, incl. privacy considerations, and any steps taken to reduce the likelihood or detection of harm (e.g., education and training, availability of a hotline)

	1	2	3	4	5	
subitem not at all important	○	○	○	○	○	essential

Your response is too large. Try shortening some answers.

Does your paper address subitem X26-iii?

Copy and paste relevant sections from the manuscript (include quotes in quotation marks "like this" to indicate direct quotes from your manuscript), or elaborate on this item by providing additional information not in the ms, or briefly explain why the item is not applicable/relevant for your study

Your answer

RESULTS

13a) For each group, the numbers of participants who were randomly assigned, received intended treatment, and were analysed for the primary outcome

NPT: The number of care providers or centers performing the intervention in each group and the number of patients treated by each care provider in each center

Your response is too large. Try shortening some answers.

Does your paper address CONSORT subitem 13a? *

Copy and paste relevant sections from the manuscript (include quotes in quotation marks "like this" to indicate direct quotes from your manuscript), or elaborate on this item by providing additional information not in the ms, or briefly explain why the item is not applicable/relevant for your study

Yes, the paper clearly addresses the first part of subitem.

Numbers Randomly Assigned:
"A total of 2,086 patients... to receive either telemedical management combined with usual care (intervention group; n=1,040) or usual care alone (control group; n=1,046)." (Abstract, Methods)

Analysed for the Primary Outcome:
"55 MACCE (5.3%) in the usual care group (n=1046) and 36 MACCE (3.5%) in the remote patient management group (n=1040)" (Results)

Received Intended Treatment (Intervention Group):

"During the trial, ...46% of the patients made inquiries." (Results)

Regarding the second part ("NPT: The number of care providers or centers..."):

Explanation: This part is not applicable in the traditional sense, as it is designed for cluster-randomized trials where providers or centers are the unit of randomization. This was a patient-randomized, single-center trial.

13b) For each group, losses and exclusions after randomisation, together with reasons

Your response is too large. Try shortening some answers.

Does your paper address CONSORT subitem 13b? (NOTE: Preferably, this is shown in a CONSORT flow diagram) *

Copy and paste relevant sections from the manuscript (include quotes in quotation marks "like this" to indicate direct quotes from your manuscript), or elaborate on this item by providing additional information not in the ms, or briefly explain why the item is not applicable/relevant for your study

The manuscript references a participant flow diagram .

Here is the relevant direct quote from the manuscript:

"Between November 2022 and June 2023, 2,086 inpatients diagnosed with CHD who underwent PCI were enrolled in the study (Figure 3). The participants were randomly assigned to two groups: the intervention group... (n=1,040), and the control group... (n=1,046)."

Reference to Flow Diagram: The manuscript explicitly references Figure 3, which is titled "Participant Flow Diagram." This is the standard and preferred method for presenting this information, as per the CONSORT guideline note.

13b-i) Attrition diagram

Strongly recommended: An attrition diagram (e.g., proportion of participants still logging in or using the intervention/comparator in each group plotted over time, similar to a survival curve) or other figures or tables demonstrating usage/dose/engagement.

	1	2	3	4	5	
subitem not at all important	○	○	○	○	○	essential

Does your paper address subitem 13b-i?

Copy and paste relevant sections from the manuscript or cite the figure number if applicable (include quotes in quotation marks "like this" to indicate direct quotes from your manuscript), or elaborate on this item by providing additional information not in the ms, or briefly explain why the item is not applicable/relevant for your study

Your answer

Your response is too large. Try shortening some answers.

Does your paper address CONSORT subitem 14a? *

Copy and paste relevant sections from the manuscript (include quotes in quotation marks "like this" to indicate direct quotes from your manuscript), or elaborate on this item by providing additional information not in the ms, or briefly explain why the item is not applicable/relevant for your study

Yes, the paper clearly addresses this CONSORT subitem.

Recruitment Period: The manuscript explicitly states that recruitment occurred "between November 2022 and June 2023".

Follow-up Period: While not explicitly stating the exact end date for follow-up, the manuscript clearly defines that the primary outcome was "assessed during the 1-year follow-up after randomization" (Methods: Outcome). Given that the last participants were enrolled in June 2023, the follow-up period would have extended until approximately June 2024.

The combination of the specific recruitment dates and the clearly defined 1-year follow-up duration provides the necessary information to understand the trial timeline, thus fully addressing this subitem.

14a-i) Indicate if critical "secular events" fell into the study period

Indicate if critical "secular events" fell into the study period, e.g., significant changes in Internet resources available or "changes in computer hardware or Internet delivery resources"

	1	2	3	4	5	
subitem not at all important	○	○	○	○	○	essential

Does your paper address subitem 14a-i?

Copy and paste relevant sections from the manuscript (include quotes in quotation marks "like this" to indicate direct quotes from your manuscript), or elaborate on this item by providing additional information not in the ms, or briefly explain why the item is not applicable/relevant for your study

Your answer

Your response is too large. Try shortening some answers.

14b) Why the trial ended or was stopped (early)

Does your paper address CONSORT subitem 14b? *

Copy and paste relevant sections from the manuscript (include quotes in quotation marks "like this" to indicate direct quotes from your manuscript), or elaborate on this item by providing additional information not in the ms, or briefly explain why the item is not applicable/relevant for your study

This item is not applicable to our study.

Explanation: The trial was not stopped early. It was conducted as planned, completing the pre-specified recruitment and follow-up periods.

Recruitment was conducted between November 2022 and June 2023.

Follow-up was completed for all participants for the pre-defined period of one year after randomization.

15) A table showing baseline demographic and clinical characteristics for each group

NPT: When applicable, a description of care providers (case volume, qualification, expertise, etc.) and centers (volume) in each group

Your response is too large. Try shortening some answers.

Does your paper address CONSORT subitem 15? *

Copy and paste relevant sections from the manuscript (include quotes in quotation marks "like this" to indicate direct quotes from your manuscript), or elaborate on this item by providing additional information not in the ms, or briefly explain why the item is not applicable/relevant for your study

Yes, the paper fully addresses the first part of this CONSORT subitem.

Direct Quote/Evidence:

"The baseline clinical and laboratory characteristics, as well as the utilization of cardiovascular medications, were comparable between the two groups (Tables 1 and 2)." (Results section)

Regarding the second part ("NPT: When applicable, a description of care providers... in each group"):

Explanation: This part is partially addressed.

"Throughout the study, four health managers served as full-time staff members at the telemedical center during daytime hours. Additionally, three registered nurses and three registered doctors were tasked with providing health education and addressing patients' inquiries..." (Results section)

15-i) Report demographics associated with digital divide issues

In ehealth trials it is particularly important to report demographics associated with digital divide issues, such as age, education, gender, social-economic status, computer/Internet/ehealth literacy of the participants, if known.

	1	2	3	4	5	
subitem not at all important	○	○	○	○	○	essential

Does your paper address subitem 15-i? *

Copy and paste relevant sections from the manuscript (include quotes in quotation marks "like this" to indicate direct quotes from your manuscript), or elaborate on this item by providing additional information not in the ms, or briefly explain why the item is not applicable/relevant for your study

[illegible]

Your response is too large. Try shortening some answers.

16) For each group, number of participants (denominator) included in each analysis and whether the analysis was by original assigned groups

16-i) Report multiple “denominators” and provide definitions

Report multiple “denominators” and provide definitions: Report N’s (and effect sizes) “across a range of study participation [and use] thresholds” [1], e.g., N exposed, N consented, N used more than x times, N used more than y weeks, N participants “used” the intervention/comparator at specific pre-defined time points of interest (in absolute and relative numbers per group). Always clearly define “use” of the intervention.

	1	2	3	4	5	
subitem not at all important	○	○	○	○	○	essential

Your response is too large. Try shortening some answers.

Does your paper address subitem 16-i? *

Copy and paste relevant sections from the manuscript (include quotes in quotation marks "like this" to indicate direct quotes from your manuscript), or elaborate on this item by providing additional information not in the ms, or briefly explain why the item is not applicable/relevant for your study

The manuscript provides some engagement metrics that serve as different "denominators" .

Here are the relevant direct quotes from the manuscript:

1: Allocated to Intervention (N=1040)
"the intervention group, which received remote patient management in addition to usual care (n=1,040)"

2: Received Materials (N=1040)

"all participants in the intervention group received health materials—either text messages or videos— and follow-up questionnaires were sent to them."

3: Engaged with Materials (81%)

"Among these patients, 81% reported having read the health messages..."

4: Responded to Questionnaires (68%)

"...while the response rate for the follow-up questionnaire was 68%."

5: Active Users Making Inquiries (46%)

"Furthermore, 46% of the patients made inquiries."

16-ii) Primary analysis should be intent-to-treat

Primary analysis should be intent-to-treat, secondary analyses could include comparing only “users”, with the appropriate caveats that this is no longer a randomized sample (see 18-i).

	1	2	3	4	5	
subitem not at all important	○	○	○	○	○	essential

Your response is too large. Try shortening some answers.

Does your paper address subitem 16-ii?

Copy and paste relevant sections from the manuscript (include quotes in quotation marks "like this" to indicate direct quotes from your manuscript), or elaborate on this item by providing additional information not in the ms, or briefly explain why the item is not applicable/relevant for your study

Your answer

17a) For each primary and secondary outcome, results for each group, and the estimated effect size and its precision (such as 95% confidence interval)

Does your paper address CONSORT subitem 17a? *

Copy and paste relevant sections from the manuscript (include quotes in quotation marks "like this" to indicate direct quotes from your manuscript), or elaborate on this item by providing additional information not in the ms, or briefly explain why the item is not applicable/relevant for your study

The paper presents results for each group for all outcomes but consistently fails to report the estimated effect sizes and their precision (confidence intervals).

Primary Outcome (MACCE) - Results for each group:

"55 MACCE (5.3%) in the usual care group and 36 MACCE (3.5%) in the remote patient management group... The difference between the two groups was statistically significant (*P=* .044)."

Missing: The absolute risk reduction (ARR = 1.8%), relative risk (RR) or hazard ratio (HR), and their corresponding 95% confidence intervals are not reported.

Your response is too large. Try shortening some answers.

17a-i) Presentation of process outcomes such as metrics of use and intensity of use

In addition to primary/secondary (clinical) outcomes, the presentation of process outcomes such as metrics of use and intensity of use (dose, exposure) and their operational definitions is critical. This does not only refer to metrics of attrition (13-b) (often a binary variable), but also to more continuous exposure metrics such as “average session length”. These must be accompanied by a technical description how a metric like a “session” is defined (e.g., timeout after idle time) [1] (report under item 6a).

	1	2	3	4	5	
subitem not at all important	○	○	○	○	○	essential

Does your paper address subitem 17a-i?

Copy and paste relevant sections from the manuscript (include quotes in quotation marks "like this" to indicate direct quotes from your manuscript), or elaborate on this item by providing additional information not in the ms, or briefly explain why the item is not applicable/relevant for your study

Your answer

17b) For binary outcomes, presentation of both absolute and relative effect sizes is recommended

Does your paper address CONSORT subitem 17b? *

Copy and paste relevant sections from the manuscript (include quotes in quotation marks "like this" to indicate direct quotes from your manuscript), or elaborate on this item by providing additional information not in the ms, or briefly explain why the item is not applicable/relevant for your study

The manuscript presents results for binary outcomes (e.g., MACCE, mortality, adherence) by reporting the proportions (percentages) in each group and the p-values. However, it does not present the recommended effect sizes.

Your response is too large. Try shortening some answers.

18) Results of any other analyses performed, including subgroup analyses and adjusted analyses, distinguishing pre-specified from exploratory

Does your paper address CONSORT subitem 18? *

Copy and paste relevant sections from the manuscript (include quotes in quotation marks "like this" to indicate direct quotes from your manuscript), or elaborate on this item by providing additional information not in the ms, or briefly explain why the item is not applicable/relevant for your study

The manuscript presents only the results of the primary, unadjusted analyses for the primary and secondary outcomes. There is no mention or presentation of Subgroup analyses. The results of pre-specified subgroup analyses will be presented in a subsequent manuscript focusing on predictors of response to the telemedical intervention.

18-i) Subgroup analysis of comparing only users

A subgroup analysis of comparing only users is not uncommon in ehealth trials, but if done, it must be stressed that this is a self-selected sample and no longer an unbiased sample from a randomized trial (see 16-iii).

	1	2	3	4	5	
subitem not at all important	○	○	○	○	○	essential

Does your paper address subitem 18-i?

Copy and paste relevant sections from the manuscript (include quotes in quotation marks "like this" to indicate direct quotes from your manuscript), or elaborate on this item by providing additional information not in the ms, or briefly explain why the item is not applicable/relevant for your study

Your answer

19) All important harms or unintended effects in each group

(for specific guidance see CONSORT for harms)

Your response is too large. Try shortening some answers.

Does your paper address CONSORT subitem 19? *

Copy and paste relevant sections from the manuscript (include quotes in quotation marks "like this" to indicate direct quotes from your manuscript), or elaborate on this item by providing additional information not in the ms, or briefly explain why the item is not applicable/relevant for your study

Presentation in Results Table:
The "Table 3. Adverse Events at 1-Year follow-up visit" provides a clear breakdown of "Any bleeding" and its BARC classifications (1, 2, and 3-5) for both the intervention and control groups.
Discussion of Harms:
The discussion section specifically interprets these findings, including a plausible explanation for the observed increase in BARC 2 bleeding.
"Our study revealed that remote patient management was associated with an increased risk of BARC 2 bleeding events... this has led to a reduction in the incidence of BARC 3-5 bleeding events."

19-i) Include privacy breaches, technical problems

Include privacy breaches, technical problems. This does not only include physical “harm” to participants, but also incidents such as perceived or real privacy breaches [1], technical problems, and other unexpected/unintended incidents. “Unintended effects” also includes unintended positive effects [2].

	1	2	3	4	5	
subitem not at all important	○	○	○	○	○	essential

Does your paper address subitem 19-i?

Copy and paste relevant sections from the manuscript (include quotes in quotation marks "like this" to indicate direct quotes from your manuscript), or elaborate on this item by providing additional information not in the ms, or briefly explain why the item is not applicable/relevant for your study

Your answer

Your response is too large. Try shortening some answers.

19-ii) Include qualitative feedback from participants or observations from staff/researchers

Include qualitative feedback from participants or observations from staff/researchers, if available, on strengths and shortcomings of the application, especially if they point to unintended/unexpected effects or uses. This includes (if available) reasons for why people did or did not use the application as intended by the developers.

	1	2	3	4	5	
subitem not at all important	○	○	○	○	○	essential

Does your paper address subitem 19-ii?

Copy and paste relevant sections from the manuscript (include quotes in quotation marks "like this" to indicate direct quotes from your manuscript), or elaborate on this item by providing additional information not in the ms, or briefly explain why the item is not applicable/relevant for your study

Your answer

DISCUSSION

22) Interpretation consistent with results, balancing benefits and harms, and considering other relevant evidence

NPT: In addition, take into account the choice of the comparator, lack of or partial blinding, and unequal expertise of care providers or centers in each group

22-i) Restate study questions and summarize the answers suggested by the data, starting with primary outcomes and process outcomes (use)

Restate study questions and summarize the answers suggested by the data, starting with primary outcomes and process outcomes (use).

	1	2	3	4	5	
[illegible]	○	○	○	○	○	[illegible]

Your response is too large. Try shortening some answers.

Does your paper address subitem 22-i? *

Copy and paste relevant sections from the manuscript (include quotes in quotation marks "like this" to indicate direct quotes from your manuscript), or elaborate on this item by providing additional information not in the ms, or briefly explain why the item is not applicable/relevant for your study

The discussion section effectively restates and answers the primary study question.

Here is the relevant analysis:

Primary Outcome Question & Answer (Addressed):

Restated Question: The discussion begins by directly addressing the main efficacy question.

"Remote medical interventional management demonstrated significant benefits in reducing the incidence of cardiac mortality and myocardial infarction over a one-year study period."

Summary of Answer: It clearly summarizes the answer suggested by the data for the primary outcome (MACCE) and its key components.

"In this randomized controlled trial, we compared ... attributed to the lower rates of cardiac death and myocardial infarction at one year."

22-ii) Highlight unanswered new questions, suggest future research

Highlight unanswered new questions, suggest future research.

	1	2	3	4	5	
subitem not at all important	○	○	○	○	○	essential

Does your paper address subitem 22-ii?

Copy and paste relevant sections from the manuscript (include quotes in quotation marks "like this" to indicate direct quotes from your manuscript), or elaborate on this item by providing additional information not in the ms, or briefly explain why the item is not applicable/relevant for your study

Your answer

Your response is too large. Try shortening some answers.

20) Trial limitations, addressing sources of potential bias, imprecision, and, if relevant, multiplicity of analyses

20-i) Typical limitations in ehealth trials

Typical limitations in ehealth trials: Participants in ehealth trials are rarely blinded. Ehealth trials often look at a multiplicity of outcomes, increasing risk for a Type I error. Discuss biases due to non-use of the intervention/usability issues, biases through informed consent procedures, unexpected events.

	1	2	3	4	5	
subitem not at all important	○	○	○	○	○	essential

Does your paper address subitem 20-i? *

Copy and paste relevant sections from the manuscript (include quotes in quotation marks "like this" to indicate direct quotes from your manuscript), or elaborate on this item by providing additional information not in the ms, or briefly explain why the item is not applicable/relevant for your study

The manuscript discusses several important limitations, some of which are typical for eHealth trials.

Here are the relevant direct quotes from the "Strengths and Limitations" section of the manuscript:

"First, the findings may not be universally applicable across all cultures, as they are specific to the Chinese context..."
"Second, data on several indicators relevant to the development of CHD, such as patients' lipid levels, glycated hemoglobin, and body mass index (BMI), were not collected during the follow-up consultations."
"Third, some data regarding lifestyle changes and medication adherence were based on self-reports from patients, which could have introduced recall bias."
"Fourth, although we employed a multi-component intervention design, we could not precisely assess the independent contributions of each component..."
"Finally, although our study demonstrated the benefits associated with telemedicine interventions, air pollution factors were excluded from our analysis."

Your response is too large. Try shortening some answers.

21) Generalisability (external validity, applicability) of the trial findings

NPT: External validity of the trial findings according to the intervention, comparators, patients, and care providers or centers involved in the trial

21-i) Generalizability to other populations

Generalizability to other populations: In particular, discuss generalizability to a general Internet population, outside of a RCT setting, and general patient population, including applicability of the study results for other organizations

	1	2	3	4	5	
subitem not at all important	○	○	○	○	○	essential

Does your paper address subitem 21-i?

Copy and paste relevant sections from the manuscript (include quotes in quotation marks "like this" to indicate direct quotes from your manuscript), or elaborate on this item by providing additional information not in the ms, or briefly explain why the item is not applicable/relevant for your study

Your answer

21-ii) Discuss if there were elements in the RCT that would be different in a routine application setting

Discuss if there were elements in the RCT that would be different in a routine application setting (e.g., prompts/reminders, more human involvement, training sessions or other co-interventions) and what impact the omission of these elements could have on use, adoption, or outcomes if the intervention is applied outside of a RCT setting.

	1	2	3	4	5	
subitem not at all important	○	○	○	○	○	essential

Your response is too large. Try shortening some answers.

Does your paper address subitem 21-ii?

Copy and paste relevant sections from the manuscript (include quotes in quotation marks "like this" to indicate direct quotes from your manuscript), or elaborate on this item by providing additional information not in the ms, or briefly explain why the item is not applicable/relevant for your study

Your answer

OTHER INFORMATION

23) Registration number and name of trial registry

Does your paper address CONSORT subitem 23? *

Copy and paste relevant sections from the manuscript (include quotes in quotation marks "like this" to indicate direct quotes from your manuscript), or elaborate on this item by providing additional information not in the ms, or briefly explain why the item is not applicable/relevant for your study

Yes, the paper clearly addresses this CONSORT subitem.

Here is the relevant direct quote from the "Ethical considerations" section of the manuscript:

"This study was approved by the Ethics Committee of The First Affiliated Hospital of the University of Science and Technology of China (Approval No. 2022-ky233) and registered with the Chinese Clinical Trial Registry (ChiCTR2200065344; URL:https://www.chictr.org.cn/showproj.html?proj=167661)."

24) Where the full trial protocol can be accessed, if available

Your response is too large. Try shortening some answers.

Does your paper address CONSORT subitem 24? *

Cite a Multimedia Appendix, other reference, or copy and paste relevant sections from the manuscript (include quotes in quotation marks "like this" to indicate direct quotes from your manuscript), or elaborate on this item by providing additional information not in the ms, or briefly explain why the item is not applicable/relevant for your study

The manuscript provides the trial registration number and a link to the registry entry, which often contains or links to the study protocol.

"This study was approved by the Ethics Committee of The First Affiliated Hospital of the University of Science and Technology of China (Approval No. 2022-ky233) and registered with the Chinese Clinical Trial Registry (ChiCTR2200065344; URL:https://www.chictr.org.cn/showproj.html?proj=167661)."

It is standard practice for registry entries like the Chinese Clinical Trial Registry to include key protocol information and sometimes a link to the full protocol document.

25) Sources of funding and other support (such as supply of drugs), role of funders

Does your paper address CONSORT subitem 25? *

Copy and paste relevant sections from the manuscript (include quotes in quotation marks "like this" to indicate direct quotes from your manuscript), or elaborate on this item by providing additional information not in the ms, or briefly explain why the item is not applicable/relevant for your study

Yes, the paper clearly addresses the first part of this CONSORT subitem by listing the sources of funding.

"This study was supported by the Joint Fund for Medical Artificial Intelligence (MAI2022Q013 and MAI2022Z001), Anhui Science and Technology Breakthrough Plan (Grant No.202423k09020030), Research Funds of Centre for Leading Medicine and Advanced Technologies of IHIM(2023IHM01063), and China Heart House-Chinese Cardiovascular Association HX fund (2022-CCA-HX-077)"

X27) Conflicts of Interest (not a CONSORT item)

Your response is too large. Try shortening some answers.

X27-i) State the relation of the study team towards the system being evaluated

In addition to the usual declaration of interests (financial or otherwise), also state the relation of the study team towards the system being evaluated, i.e., state if the authors/evaluators are distinct from or identical with the developers/sponsors of the intervention.

	1	2	3	4	5	
subitem not at all important	○	○	○	○	○	essential

Does your paper address subitem X27-i?

Copy and paste relevant sections from the manuscript (include quotes in quotation marks "like this" to indicate direct quotes from your manuscript), or elaborate on this item by providing additional information not in the ms, or briefly explain why the item is not applicable/relevant for your study

Your answer

About the CONSORT EHEALTH checklist

As a result of using this checklist, did you make changes in your manuscript? *

- ○ yes, major changes
- ◉ yes, minor changes
- ○ no

What were the most important changes you made as a result of using this checklist?

Your answer

Your response is too large. Try shortening some answers.

How much time did you spend on going through the checklist INCLUDING making changes in your manuscript *

2-3 hours for the initial comprehensive review and 1-2 hours for implementing the changes, totaling 3-5 hours

As a result of using this checklist, do you think your manuscript has improved? *

- (•) yes
- ( ) no
- ( ) Other:

Would you like to become involved in the CONSORT EHEALTH group?

This would involve for example becoming involved in participating in a workshop and writing an "Explanation and Elaboration" document

- ( ) yes
- ( ) no
- ( ) Other:

Any other comments or questions on CONSORT EHEALTH

Your answer

Your response is too large. Try shortening some answers.

STOP - Save this form as PDF before you click submit

To generate a record that you filled in this form, we recommend to generate a PDF of this page (on a Mac, simply select "print" and then select "print as PDF") before you submit it.

When you submit your (revised) paper to JMIR, please upload the PDF as supplementary file.

Don't worry if some text in the textboxes is cut off, as we still have the complete information in our database. Thank you!

Final step: Click submit !

Click submit so we have your answers in our database!

Submit

Clear form

Never submit passwords through Google Forms.

This content is neither created nor endorsed by Google. - Contact form owner - Terms of Service - Privacy Policy

Does this form look suspicious? Report

Google Forms

Your response is too large. Try shortening some answers.

Your response is too large. Try shortening some answers.
